# Meta-analysis of gene expression profiles in breast cancer: toward a unified understanding of breast cancer subtyping and prognosis signatures

**DOI:** 10.1186/bcr2124

**Published:** 2008-07-28

**Authors:** Pratyaksha Wirapati, Christos Sotiriou, Susanne Kunkel, Pierre Farmer, Sylvain Pradervand, Benjamin Haibe-Kains, Christine Desmedt, Michail Ignatiadis, Thierry Sengstag, Frédéric Schütz, Darlene R Goldstein, Martine Piccart, Mauro Delorenzi

**Affiliations:** 1 Swiss Institute of Bioinformatics, 'Batiment Genopode', University of Lausanne, 1015 Lausanne, Switzerland; 2 Translational Research and Medical Oncology Unit, Université Libre de Bruxelles, Institut Jules Bordet, 121 Boulevard de Waterloo, 1000 Brussels, Belgium; 3 National Centers for Competence in Research, Molecular Oncology, Swiss Institute for Experimental Cancer Research, Ch. des Boveresses 155, 1066 Epalinges, Switzerland; 4 DNA Array Facility, Center for Integrative Genomics, 'Batiment Genopode', University of Lausanne, 1015 Lausanne, Switzerland; 5 Machine Learning Group, Université Libre de Bruxelles, boulevard du Triomphe, CP212, 1050 Bruxelles, Belgium; 6 Institut de Mathématiques, Ecole Polytechnique Fédérale de Lausanne, 1015 Lausanne, Switzerland

## Abstract

**Introduction:**

Breast cancer subtyping and prognosis have been studied extensively by gene expression profiling, resulting in disparate signatures with little overlap in their constituent genes. Although a previous study demonstrated a prognostic concordance among gene expression signatures, it was limited to only one dataset and did not fully elucidate how the different genes were related to one another nor did it examine the contribution of well-known biological processes of breast cancer tumorigenesis to their prognostic performance.

**Method:**

To address the above issues and to further validate these initial findings, we performed the largest meta-analysis of publicly available breast cancer gene expression and clinical data, which are comprised of 2,833 breast tumors. Gene coexpression modules of three key biological processes in breast cancer (namely, proliferation, estrogen receptor [ER], and HER2 signaling) were used to dissect the role of constituent genes of nine prognostic signatures.

**Results:**

Using a meta-analytical approach, we consolidated the signatures associated with ER signaling, ERBB2 amplification, and proliferation. Previously published expression-based nomenclature of breast cancer 'intrinsic' subtypes can be mapped to the three modules, namely, the ER^-^/HER2^- ^(basal-like), the HER2^+ ^(HER2-like), and the low- and high-proliferation ER^+^/HER2^- ^subtypes (luminal A and B). We showed that all nine prognostic signatures exhibited a similar prognostic performance in the entire dataset. Their prognostic abilities are due mostly to the detection of proliferation activity. Although ER^- ^status (basal-like) and ERBB2^+ ^expression status correspond to bad outcome, they seem to act through elevated expression of proliferation genes and thus contain only indirect information about prognosis. Clinical variables measuring the extent of tumor progression, such as tumor size and nodal status, still add independent prognostic information to proliferation genes.

**Conclusion:**

This meta-analysis unifies various results of previous gene expression studies in breast cancer. It reveals connections between traditional prognostic factors, expression-based subtyping, and prognostic signatures, highlighting the important role of proliferation in breast cancer prognosis.

## Introduction

Breast cancer is the disease most extensively studied by gene expression profiling of primary tumors from patient populations [1-21]. Despite this effort, the research results are still fragmented. Disparate signatures have been proposed, either directly from breast cancer expression profiles [10-12,18,19,21,22] or translated from model systems [1,23,24], with little agreement in the constituent genes. Fan and colleagues [25] recently compared the prognostic ability of the intrinsic subtypes and four prognostic signatures in 295 patients. They noted concordance in the risk classification, which suggests potential equivalence between some of these signatures. However, these signatures have been examined in only one dataset and the study did not fully elucidate how the different genes were related to one another nor did it examine the contribution of well-known biological processes of breast cancer tumorigenesis to their prognostic performance.

To address these issues, we undertook the largest meta-analysis of publicly available gene expression and clinical data, which are comprised of 2,833 breast tumors [1-21]. We used the concept of 'coexpression' modules (comprehensive lists of genes with highly correlated expression) associated with important biological processes in breast cancer to reveal the common thread connecting molecular subtyping and several prognostic signatures. Their prognostic values, adjusted for the conventional clinicopathological variables, were studied in a database of 2,833 patients with breast cancer in order to arrive at solid conclusions. Finally, we went a step further to characterize the constituent genes of these signatures and to study how they contribute to their prognostic power.

## Materials and methods

Detailed descriptions of the methods can be found in Additional data file 1. A brief summary is outlined here.

### Preparation of expression data

**Figure 1:**
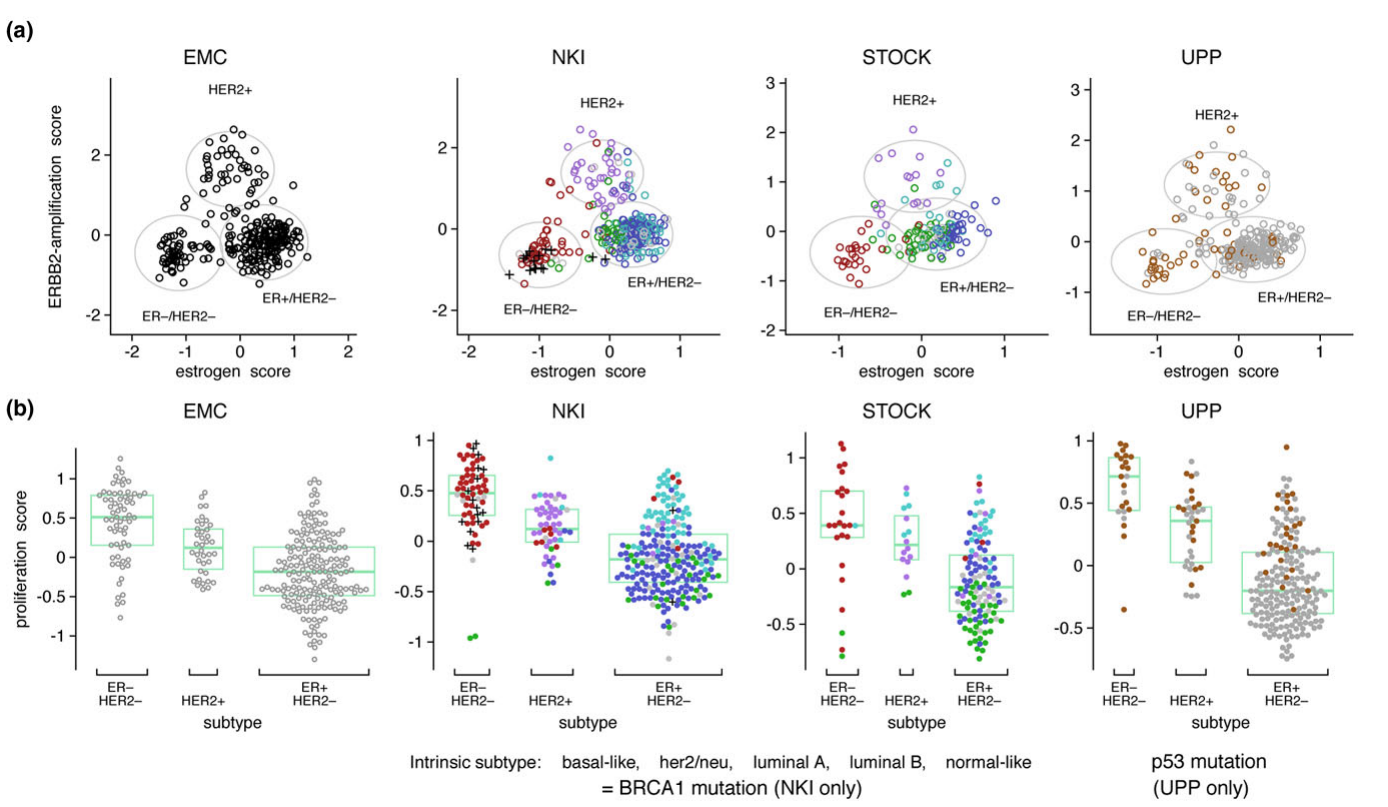
**Breast tumor characterization using module scores**. **(a) **Joint distribution between the estrogen and ERBB2 amplification scores in example datasets. Clusters are identified by Gaussian mixture models with three components. The ellipses correspond to the 95% cumulative probability around the cluster centers. The clusters are designated as tumor types ER^-^/ERBB2^-^, HER2^+^, and ER^+^/HER2^-^. HER2^+ ^tumors show intermediate estrogen scores. **(b) **Dot histograms showing dependence of proliferation score on the subtypes. The median and quartiles for each group are shown by the box plot. ER^-^/ERBB2^- ^and HER2^+ ^tumors show high proliferation scores, whereas ER^+^/HER2^- ^tumors show a wide range of proliferation scores. The distributions of the intrinsic subtypes (colored dots), BRCA1 mutations, and p53 mutations are shown in datasets where they are available. ER, estrogen receptor.

We collected publicly available datasets from journal articles and repositories such as Gene Expression Omnibus (GEO) and ArrayExpress, selecting those with a medium to large sample size (Table 1). Since publications sometimes used the same patients, datasets with unique patients were introduced (identified by the 'dataset symbols' in Table 1) by merging some original datasets or removing redundant patients. The collection includes datasets produced on whole-genome microarrays, small diagnostic arrays, and reverse transcription-polymerase chain reaction panels, totaling 2,833 expression profiles. Hybridization probes were mapped to Entrez GeneID [26] through sequence alignment against RefSeq mRNA in the (NM) subset, similar to the approach of Shi and colleagues [27], using RefSeq version 21 (2007.01.21) and Entrez database version 2007.01.21. When multiple probes were mapped to the same GeneID, the one with the highest variance in a particular dataset was selected to represent the GeneID. The numbers of distinct GeneIDs obtained for each dataset are shown in Table 1. The normalized, log-transformed expression measures as published by the original studies were used. Meta-analyses were performed on the union of all 17,198 genes. Summary statistics of absent genes were considered as missing values. Summaries of the availability and compositions of important clinical variables for each dataset are shown in Figure 1 of Additional data file 2.

**Table 1:** Publicly available gene expression data from breast cancer studies

Dataset symbol	Number of arrays	Institution	Reference(s)	Platform	Data source	Number of GeneIDs
Genomic platforms						
NKI	337	Nederlands Kanker Instituut (Amsterdam, The Netherlands)	[19,20]	Agilent	Author's website	13,120
EMC	286	Erasmus Medical Center (Rotterdam, The Netherlands)	[21]	Affymetrix U133A	GEO: GSE2034	11,837
UPP	249	Karolinksa Institute (Uppsala, Sweden)	[3,11]	Affymetrix U133A,B	GEO: GSE4922	15,684
STOCK	159	Karolinska Institute (Stockholm, Sweden)	[3,13]	Affymetrix U133A,B	GEO: GSE1456	15,684
DUKE	171	Duke University (Durham, NC, USA)	[8]	Affymetrix U95Av2	Author's website	8,149
UCSF	161 + 8	University of California at San Francisco (USA)	[9]	cDNA	Author's website	6,178
UNC	143 + 10	University of North Carolina (Chapel Hill, NC, USA)	[7]	Agilent HuA1	Author's website	13,784
NCH	135	Nottingham City Hospital (Nottingham, UK)	[12]	Agilent HuA1	AE: E-UCON-1	13,784
STNO	115 + 7	Stanford University (Palo Alto, CA, USA)/Norwegian Radium Hospital (Oslo, Norway)	[16]	cDNA	Author's website	5,614
JRH1	99	John Radcliffe Hospital (Oxford, UK)	[17]	cDNA	Journal's website	4,112
JRH2	61	John Radcliffe Hospital	[18]	Affymetrix U133A	GEO: GSE2990	11,837
MGH	60	Massachusetts General Hospital (Boston, MA, USA)	[10]	Agilent	GEO: GSE1379	11,421
expO	239	International Genomic Consortium	[41]	Affymetrix U133v2	GEO: GSE2109	16,634
TGIF1	49	EORTC trial 10994	[5]	Affymetrix U133A	GEO: GSE1561	11,837
BWH	40 + 7	Brigham and Women's Hospital (Boston, MA, USA)	[14]	Affymetrix U133v2	GEO: GSE3744	16,634
Small diagnostic platforms						
TRANSBIG	253	TRANSBIG Consortium	[2]	Agilent	AE: E-TABM-77	1,052
EMC2	180	Erasmus Medical Center	[6]	Affymetrix (custom)	GSE3453	86
HPAZ	96	Hospital La Paz (Madrid, Spain)	[4]	RT-PCR	Appendix of [4]	61
Total	2,865 = 2,833 carcinomas + 32 nonmalignant breast tissues		Number of the union of all GeneIDs:			17,198
			Number of GeneIDs common to genomic platforms:			1,963

Datasets UNC, STNO, UCSF, and BWH include a small number of normal breast or fibroadenoma tissue samples. AE, ArrayExpress (accession); Affymetrix, Affymetrix, Inc., Santa Clara, CA, USA; Agilent, Agilent Technologies, Inc., Santa Clara, CA, USA; EORTC, European Organization for Research and Treatment of Cancer; GEO, Gene Expression Omnibus (accession); RT-PCR, reverse transcription-polymerase chain reaction.

### Identifying coexpression modules

The expression levels of the prototype genes on the log_2 _scale were used as explanatory variables in multiple regression with the Gaussian error model, using the following equation (gene symbols stand for their log expression, and coefficients are omitted for clarity):

*Y*_*i *_= *ESR*1 + *ERBB*2 + *AURKA*,

where the response variable *Y*_*i *_is the expression of gene *i*. This model is fitted separately for each gene *i *in the array. The association between gene *i *and prototype *j *(in the presence of or conditional on all other prototypes) is tested using the *t *statistic for each coefficient. Because the *t *statistics for different datasets have different degrees of freedom, we put them all on the same scale by transforming them to the corresponding cumulative probabilities and then to *z *scores using the inverse standard normal cumulative distribution function. The *z *scores were combined meta-analytically across datasets using the 'inverse normal method'. The linear model above was fitted separately to each gene in each dataset, and the *z *scores were combined meta-analytically over multiple studies using the inverse normal method [28]. To select genes that are most strongly associated with the prototypes, we use a stringent criterion of |*z*| ≥ 16, which is well above |*z*| ≈ 5 that corresponds to a Bonferroni-corrected *P *value of 0.05.

### Module scores

For a specific dataset, the module score is computed for each sample as

$$module\;score=\sum_{i}w_{i}x_{i}/\sum_{i}\left|w_{i}\right|,$$

where *x*_*i *_is the expression of a gene in the module that is present in the dataset's platform. *w*_*i *_is either +1 or -1, depending on the sign of the *z *score of the association with the prototypes.

### Clustering and multimodality tests

To cluster the tumors based on the ESR1 and ERBB2 module scores, Gaussian mixture models [29] with equal and diagonal variance for all clusters were fitted. For testing multimodality, we used the likelihood ratio test statistics between the fitted model for the tested number of components, *k*, versus the alternative model with *k *- 1 components. The statistical significance of the number of components was assessed by parametric bootstrapping. Each tumor was automatically classified as estrogen receptor-negative (ER^-^)/HER2^+^, HER2^+^, or ER^+ ^using the maximum posterior probability of membership in the clusters.

### Survival analysis

Survival curves and 5-year survival rates in forest plots were based on Kaplan-Meier estimates, with the Greenwood method used for computing the 95% confidence intervals [30]. Hazard ratios between two groups were calculated using Cox regression. Stratified Cox regression was used to compute total hazard ratios in forest plots and multivariate analysis, using the dataset as the stratum indicator, thus allowing for different baseline hazard functions between cohorts. Cox regression was also used to compute gene-by-gene associations with survival, treating the log expression measures as continuous explanatory variables. The gene-wise *z *scores were combined across datasets using the inverse normal meta-analytical methods. Distant relapse-free survival (DRFS) was considered as an event for our survival analysis, which includes distant recurrence, death from breast cancer, death from a cause other than breast cancer, and death from an unknown cause.

## Results

### Prototype-based coexpression module analysis

To perform this meta-analysis including several heterogeneous datasets and different microarray platforms, we used the concept of coexpression modules. To identify these modules, we applied a supervised approach whereby three 'prototype' genes representing three key biological processes in breast cancer (namely, proliferation, ER, and HER2 amplification signaling) were selected. The genes chosen as their prototypes were, respectively, *ESR1*, *ERBB2*, and *AURKA *(aurora-related kinase 1, also known as *STK6 *or *STK15*).

**Figure 2:**
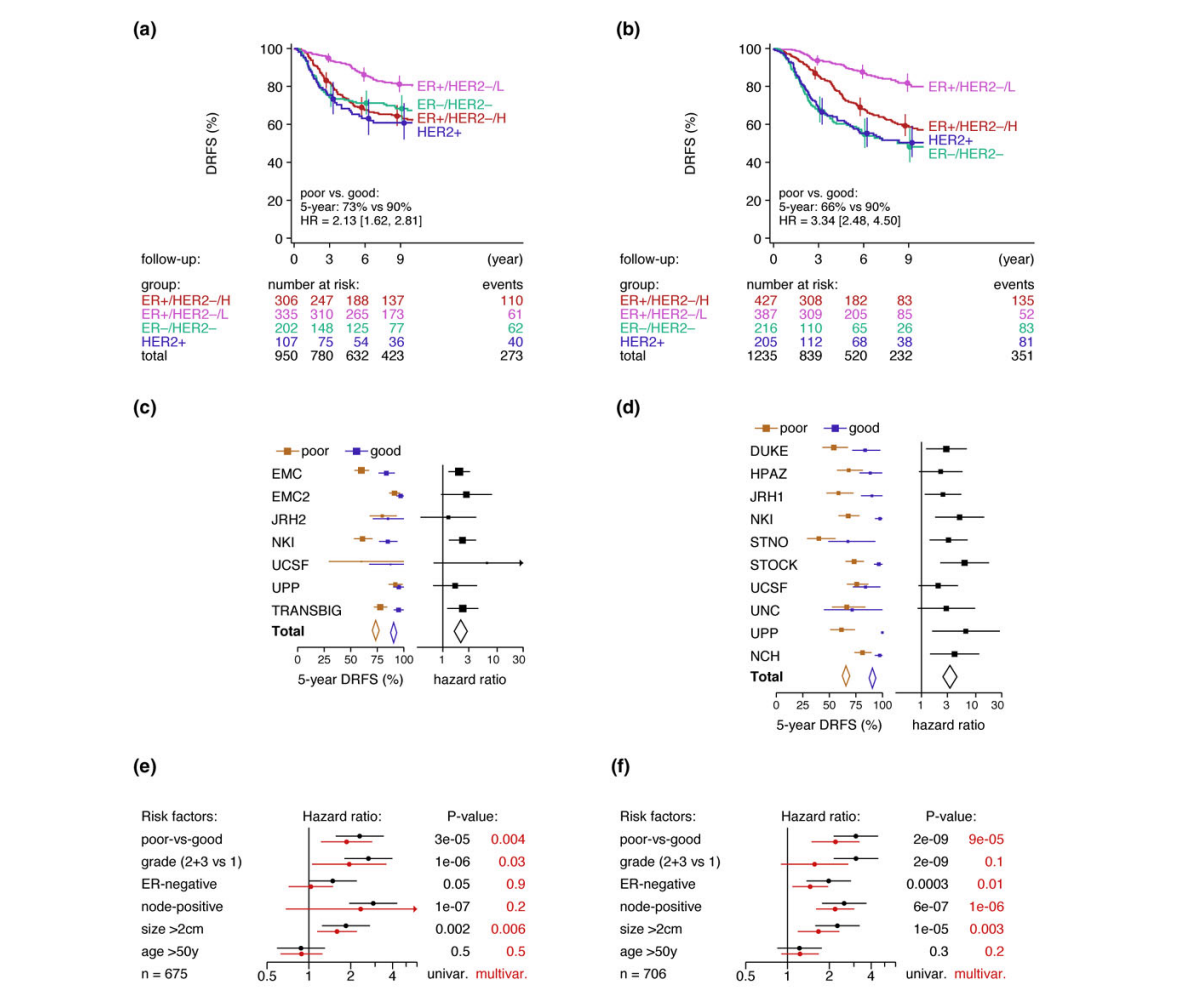
Survival analysis of groups based on module scores. Kaplan-Meier analysis for distant relapse-free survival (DRFS) of systemically untreated **(a) **and treated **(b) **patient groups. The ER^+ ^subgroup is split into ER^+^/HER2^-^/L and ER^+^/HER2^-^/H (low and high proliferation, respectively). Vertical bars on the curves are 95% confidence intervals for the Kaplan-Meier survival estimates. Forest plot representation of the 5-year survival estimates and hazard ratios for DRFS of individual datasets in the systemically untreated **(c) **and treated **(d) **populations. The length of horizontal bars and the width of the diamonds of the 'Total' correspond to 95% confidence intervals. Missing bars are unavailable data. Multivariate analysis representation in which all the variables are available in systemically untreated **(e) **and treated **(f) **patients. ER, estrogen receptor; HR, hazard ratio.

Using the meta-analysis scheme described above, we were able to identify genes whose expression was significantly associated with each chosen prototype (Additional data file 3). The coexpression patterns of the genes are shown by heatmaps in Figure 2 of Additional data file 2. Each module contains highly correlated or anticorrelated genes, as shown by the vertical color patterns. The annotation of the modules shows that they correspond well to the expected biological processes, as many ER-related, HER2-related, and proliferation genes were included in the ER and HER2 signaling and proliferation modules, respectively. For our further analysis, the correlated gene expression measures in a module (which provide redundant information) are averaged into a single number called a 'module score'.

### Module scores for tumor subtyping

To automatically assign these large numbers of tumors into the subtypes according to the given module, we applied the Gaussian mixture models [29] to the module scores of the three processes. Only three natural clusters, based on multimodality tests, can be identified. The ER and HER2 module scores were bimodally distributed, but the proliferation module was not. Furthermore, the combination of the ER and HER2 module scores does not produce four clusters that would have been observed if the scores were independent (Figure 1a). Instead, ERBB2^+ ^tumors showed an intermediate level of ER module values, and we therefore did not consider the distinction of ERBB2^+ ^into ER^+ ^or ER^- ^to be supported by continuous value gene expression levels. We will refer to three groups as ER^-^/HER2^-^, HER2^+^, and ER^+^/HER2^- ^tumors, which correspond roughly to the intrinsic subtypes of basal-like, *her2*, and combined luminal A/B subtypes, respectively, as defined by the Stanford group [15].

Concerning proliferation, Figure 1b shows that, while ER^-^/HER2^- ^and HER2^+ ^tumors have mostly high proliferation scores, ER^+^/HER2^- ^tumors display a wide range of values, encompassing the low values of normal breast tissue (see dataset UNC) and the high values typical for ER^-^/HER2^- ^and HER2^+ ^tumors. For our further analysis, we denote the ER^+^/HER2^- ^low- and high-proliferation tumors as ER^+^/HER2^-^/L and ER^+^/HER2^-^/H, corresponding to the luminal A and B subdivisions of the intrinsic subtypes, respectively. Interestingly, we did not see natural clustering (bimodality) in the distribution of proliferation scores as was the case with the ER and ERBB2 modules.

The relationship between module scores and some gene mutations could also be examined. Almost all BRCA1-mutated tumors are confined to ER^- ^tumors (Figure 1b), confirming the hypothesis that ER^- ^('basal-like') tumors are phenocopies of BRCA1-mutated tumors [14]. This is also supported by the strong overexpression of LMO4, a suppressor of BRCA1 function [31], in ER^- ^tumors. p53 mutations may appear in the three subtypes, but mostly confined to the highly proliferative tumors. It is not clear whether their association with ER^-^/HER2^- ^and HER2^+ ^tumors is related to the pathways of these receptors or is merely an indirect effect of the mutations' association with proliferation.

### Prognostic value of the molecular subtypes according to the module scores

The attractiveness of gene expression prognostic signatures for clinical applications comes from their ability to identify a group of patients with a good survival rate that is acceptable to spare patients from aggressive chemotherapy. Here, we investigated whether classifications based on the easily interpretable module scores could achieve such clinical relevance.

Figure 2 shows a Kaplan-Meier analysis for the DRFS of systemically untreated (Figure 2a) patients and those treated (Figure 2b) with adjuvant chemotherapy and/or endocrine therapy with available clinical information, according to four main subtypes based on the module scores. The ER^+^/HER2^-^/L subtype showed a much better DRFS than the three others in both untreated and treated populations, with 90% of patients alive at 5 years of follow-up. Because there is no statistical difference in survival between the ER^-^, HER2^+^, and ER^+^/HER2^-^/H subtypes and because the risk of recurrence for patients in these groups is clinically still too high, we pooled them into the 'poor' prognosis group, in contrast to the 'good' ER^+^/HER2^-^/L subtype, for further survival analysis. The consistency of the prognostic value across datasets is demonstrated by the forest plots in Figures 2c and 2d, where the results of the analysis of individual datasets are concisely summarized by the 5-year survival estimates and hazard ratios between the 'good' and 'poor' groups. Interestingly, the 'good' prognosis group showed a better DRFS than the 'poor' prognosis group in both untreated and systemically treated populations.

The interactions between the module-based risk groups and conventional clinicopathological prognostic variables are tested in multivariable Cox regression analysis for DRFS in both untreated (Figure 2e) and treated (Figure 2f) populations. The module-based classification added a strong prognostic effect over all other clinical factors. Confirming previous studies [18,32], the effect of histological grade is much reduced and can be explained by the refinement of intermediate grade into two groups with very different survival rates. Interestingly, lymph node status and tumor size remain as independent prognostic factors.

### Dissecting gene expression prognostic signatures according to the module scores

Although Fan and colleagues [25] noted the similarity of the performance and patient classifications of the intrinsic subtypes and four prognostic signatures on the same dataset, they did not provide a biological rationale for this finding. In our study, we performed more detailed and extensive analysis to better understand how disparate gene lists may give rise to potentially equivalent prognostic signatures.

Using our meta-analytical approach, we first sought to identify individual genes that were associated with survival by calculating the meta-analytical *z *scores of gene-by-gene Cox regression. To gain further insight into the biological significance of these prognostic genes, we investigated their correlation with the coexpression module prototypes. We were able to identify 524 genes that were significantly associated with survival, even under a stringent Bonferroni multiple testing correction (data not shown). Of the 524 genes, 71% were strongly coexpressed with proliferation, 26% with ER, and 2.2% with ERBB2 prototypes, highlighting the importance of proliferation-related genes for prognostication in breast cancer.

**Figure 3:**
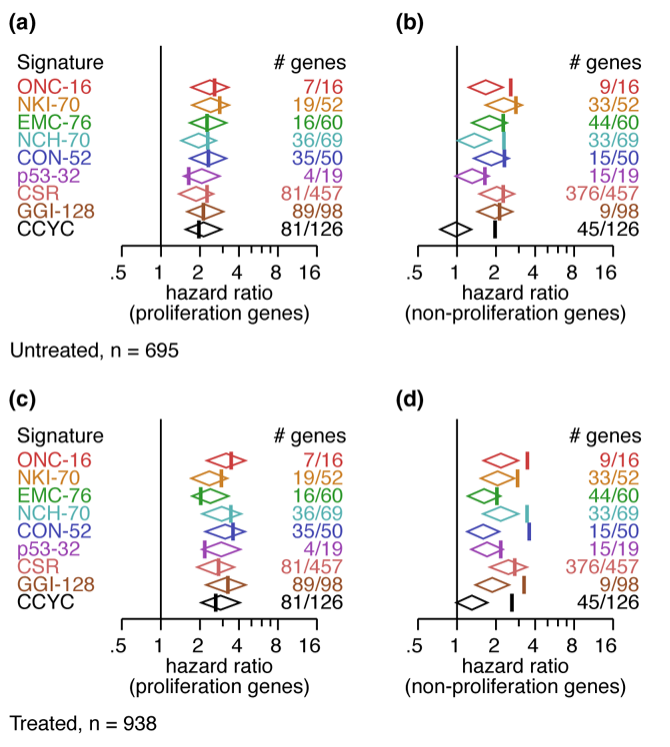
Signature comparison. The prognostic performance of the signatures is compared by the forest plots of hazard ratio and plotted as vertical color bars for comparison. Most signatures show similar performance. Prognostic performance for distant relapse-free survival (DRFS) of the signatures using partial signatures containing only proliferation genes in the untreated **(a) **and treated **(c) **populations. The performance of most signatures is not degraded; in fact, it is improved for p53-32. Prognostic performance for DRFS of the signatures using partial signatures containing nonproliferation genes in the untreated **(b) **and treated **(d) **populations.

A similar analysis was performed with respect to several published prognostic signatures (Table 2). Indeed, many of the genes included in these signatures were confirmed to be individually prognostic in the whole dataset collection (Figure 3 of Additional data file 2). Interestingly, many of these individually prognostic genes were also highly correlated with the proliferation module prototype and not with the other two modules, suggesting that proliferation may be the common driving force of several prognostic signatures.

**Table 2:** Prognostic signatures

Signature symbol	Reference	Associated variables in gene selection procedure	Number of genes	
			Original probes	Mapped to geneID

ONC-16	[42]	Biological knowledge; refined by patient outcome	16	16
NKI-70	[19]	Patient outcome	70	52
EMC-76	[21]	Patient outcome, stratified by estrogen receptor status	60 + 16	48 + 12
NCH-70	[12]	Patient outcome	70	69
CON-52	[43]	Patient outcome, consensus	52	50
p53-32	[11]	p53 mutation	32	19
CSR	[24]	Fibroblast core serum response	512	457
GGI-128	[18]	Histological grade	128	98
CCYC	[44]	Periodic expression in cell cycle progression	NA	126

NA, not applicable.

**Figure 4:**
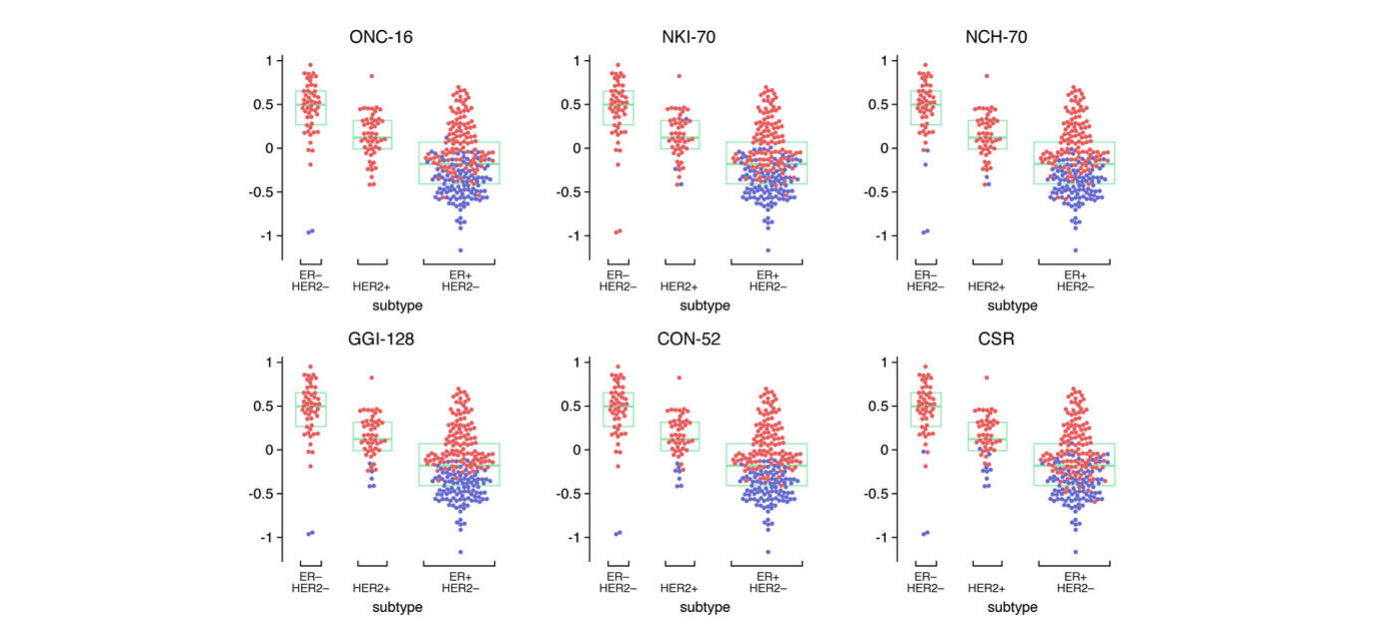
Patient classifications made by example signatures applied to representative datasets, showing that the different signatures are essentially detecting as low-risk the low-proliferation subset of ER^+^/ERBB2^- ^tumors. ER, estrogen receptor.

To further demonstrate our hypothesis, we divided each signature into two 'partial signatures': one with only proliferation genes and the other with the complementary nonproliferation genes (Figure 3; see Figure 4 of Additional data file 2 for detailed analysis). Interestingly, when only proliferation genes were used, the overall performance was not degraded; in fact, it even improved for some signatures (p53-32) in both untreated (Figure 3a) and treated (Figure 3c) populations. In contrast, the nonproliferation partial signatures typically showed degraded performance (Figures 3b and 3d). These results show that proposed signatures may contain genes that are unnecessary or even detrimental to their performance. These results thus extend the findings of Fan and colleagues [25] to a much larger sample size and for several additional signatures, revealing for the first time the importance of proliferation genes as a common driving force behind the performance of all of the prognostic signatures studied in this investigation.

Finally, the relationship between prognostic signatures and the molecular classification based on the coexpression modules was investigated by looking at the risk classifications on the plots of proliferation scores versus the molecular subtypes shown in Figure  4 (see Figure 4 of Additional data file 2 for analysis on all datasets). Most signatures identified the low-proliferation subset of ER^+^/HER2^- ^tumors as low-risk, whereas almost all high-proliferation ER^+^, ER^-^/HER2^-^, and HER2^+ ^tumors were classified as high-risk. These results suggest that these prognostic signatures function mostly by identifying tumors that have high expression of proliferation genes, regardless of the subtyping based on ER or HER2. They still correctly classify ER^-^/HER2 and HER2^+ ^as high-risk by virtue of elevated expression of proliferation genes.

## Discussion

Several breast cancer studies have generated a large number of arrays with complex genomic data, and an initial effort was made to compare the prognostic performance of the intrinsic subtypes and four signatures in one dataset [25]. In the present meta-analysis, we analyzed data from 2,833 patients to have the power to address the following questions: How are different signatures related with respect to prognostication? Should clinical, pathological, and currently used biomarkers be integrated into this process? What is the role of individual genes in a signature, and what is their biological meaning?

Using our meta-analytical approach, we confirmed the presence of four stable breast cancer molecular subtypes as originally reported by Perou and colleagues [33], whereas the normal-like subtype was not verified. Both ER^-^/HER2^- ^and HER2^+ ^subtypes were characterized by high proliferation, whereas the ER^+^/HER2^- ^subtype was divided into low- and high-proliferation tumors with different clinical outcomes. The widely observed prognostic powers of ER and HER2 are therefore only indirect effects.

Furthermore, the above results have important clinical implications since they suggest that all investigated prognostic signatures are equivalent. This will be further validated when the results from the currently accruing MINDACT (Microarray in Node-Negative Disease May Avoid Chemotherapy) [34] and TAILORX (Trial Assigning IndividuaLized Options for Treatment [Rx]) [35] trials are reported. For the ER^-^/HER2^- ^and HER2^+ ^patients, new prognostic signatures, which do not rely on proliferation genes, are urgently needed. Initial efforts to improve prognosis in the above high-risk subgroups were recently reported [36,37].

Moreover, rather than treating the signatures as black boxes, the connection to the breast cancer biology has been elucidated. Using this approach, we demonstrated that several previously reported prognostic signatures, despite the disparity in their gene lists, carry similar information with regard to prognostication. Although it may be argued that microarray measurements are merely alternative ways to monitor well-known processes such as proliferation, ER, or HER2 signaling, their results are not perfectly concordant with conventional variables. For example, although the proliferation module score and histological grade both aim to measure cell proliferation, the former is more informative [18]. We observed that HER2^+ ^tumors showed intermediate ER module activity, which is not obvious from the traditional ER and HER2 status using conventional assays. These examples suggest that the assessment of several genes from a coexpression module may provide a more accurate quantification of a whole transcriptional process than using single-gene markers or histopathological variables.

Blamey [38] distinguished independent prognostic factors into those related to the extent of tumor progression (such as lymph node status and tumor size) and those related to a tumor's intrinsic aggressiveness (such as histological grade and mitotic rate) and found only that the prognostic roles of many markers, such as ER, progesterone receptor, and p53, were overshadowed by histological grade. Our results confirmed these observations, as proliferation genes are even better indicators of tumor grade [18]. The proliferation score already contains the poor prognosis information attributable to various sources: for example, ERBB2 amplification (with or without BRCA1 mutation), p53 mutation, or yet unknown factors specifically affecting half of ER^+ ^(luminal) tumors. We still see the prognostic effect of lymph node status and tumor size, suggesting that they influence outcome through their own independent paths.

Despite the lack of direct prognostic impact of ER and ERBB2 genes, the coexpression modules for these processes that we identified are still useful. Genes in the proliferation module are already targeted by several chemotherapeutic agents, but less harmful drugs are more desirable. ER^+^/HER2^- ^tumors are treatable to some extent by hormone therapy [39] (targeting ESR1 signaling), and HER2^+ ^tumors by trastuzumab [40] (targeting ERBB2). However, drugs specifically targeting ER^-^/HER2^- ^tumors have not yet been established. Furthermore, the fact that many breast tumors remain unresponsive to existing drugs warrants further searches for alternative targets, possibly compensatory genes in the same pathway. Our analysis provides lists of genes coexpressed with these two processes, and these lists should be more stable than previously published ones because they are identified from a large data collection from multiple platforms.

Finally, we have also shown that using coexpression modules is a versatile tool for unifying apparently disparate results. Although coexpression does not imply direct physical interaction, the highly correlated genes in a module can be considered surrogate markers of one another and of the same underlying transcriptional process. Consequently, newly published signatures in the future can be perceived in the light of well-known modules, and a new, equivalently prognostic set of markers can be devised based on subsets of these lists.

## Conclusion

In summary, this study objectively evaluates several published signatures in independent cohorts from diverse microarray platforms and unifies results of previous gene expression studies in breast cancer. With respect to clinical application, we revealed connections and equivalence between traditional prognostic factors, expression-based subtyping, and prognostic signatures and provided evidence that these signatures should be tested for their ability to spare adjuvant chemotherapy mainly in the low-proliferation subgroup of patients with ER^+ ^tumors. With respect to disease biology, we consolidated the gene lists of the major processes, providing more reliable candidates for biomarkers and therapeutic targets than those produced by single-dataset studies. Finally, we provided a new methodological framework, also applicable to other diseases, for using heterogeneous microarray datasets to uncover consistent biological relationships and to consolidate proposed signatures.

## Abbreviations

DRFS = distant relapse-free survival; ER = estrogen receptor.

## Competing interests

CS, MD, and MP are named inventors on a patent application for the genomic grade signature used in this study. The other authors declare that they have no competing interests.

## Authors' contributions

PW helped to design the overall study, compile and curate the datasets, design the statistical approaches, perform the computational analysis, and develop the prototype genes and biological interpretation. CS helped to design the overall study and provide expertise in clinical breast oncology. PW and CS contributed equally to this work. MD helped to design the overall study, design the statistical approaches, and perform the computational analysis. SK and TS helped to compile and curate the datasets. DRG helped to design the statistical approaches. SP, FS, BH-K, and CD helped to perform the computational analysis. PF helped to develop the prototype genes and biological interpretation. MP and MI helped to provide expertise in clinical breast oncology. All authors contributed to the preparation of the manuscript and read and approved the final manuscript.

## Supplementary Material

Additional file 1 Supplementary methods. Supplementary methods including the following sections: 'Probe annotation and gene matching', 'Preprocessing of expression values', 'Identifying coexpression modules', 'Module scores', 'Clustering and multimodality tests', 'Survival analysis', 'Cell-cycle periodicity' and 'Cross-platform applications of signatures'. Click here for file

Additional file 2 Supplementary results. Supplementary Results including Supplementary Figures 1–5 and the results from the 'Combined prediction by pairs of signatures' (Supplementary Figure 6). Click here for file

Additional file 3 ESR1, ERBB2 and proliferation coexpression modules. The spreadsheet describes the ESR1, ERBB2 and proliferation (AURKA) coexpression modules. The columns are described in the first lines starting by '#' in the text file. Click here for file

## References

[B1] Bild AH, Yao G, Chang JT, Wang Q, Potti A, Chasse D, Joshi MB, Harpole D, Lancaster JM, Berchuck A, Olson JA, Marks JR, Dressman HK, West M, Nevins JR (2006). Oncogenic pathway signatures in human cancers as a guide to targeted therapies. Nature.

[B2] Buyse M, Loi S, van't Veer L, Viale G, Delorenzi M, Glas AM, d'Assignies MS, Bergh J, Lidereau R, Ellis P, Harris A, Bogaerts J, Therasse P, Floore A, Amakrane M, Piette F, Rutgers E, Sotiriou C, Cardoso F, Piccart MJ, TRANSBIG Consortium (2006). Validation and clinical utility of a 70-gene prognostic signature for women with node-negative breast cancer. J Natl Cancer Inst.

[B3] Calza S, Hall P, Auer G, Bjohle J, Klaar S, Kronenwett U, Liu ET, Miller L, Ploner A, Smeds J, Bergh J, Pawitan Y (2006). Intrinsic molecular signature of breast cancer in a population-based cohort of 412 patients. Breast Cancer Res.

[B4] Espinosa E, Vara JA, Redondo A, Sanchez JJ, Hardisson D, Zamora P, Pastrana FG, Cejas P, Martinez B, Suarez A, Calero F, Barón MG (2005). Breast cancer prognosis determined by gene expression profiling: a quantitative reverse transcriptase polymerase chain reaction study. J Clin Oncol.

[B5] Farmer P, Bonnefoi H, Becette V, Tubiana-Hulin M, Fumoleau P, Larsimont D, Macgrogan G, Bergh J, Cameron D, Goldstein D, Duss S, Nicoulaz AL, Brisken C, Fiche M, Delorenzi M, Iggo R (2005). Identification of molecular apocrine breast tumours by microarray analysis. Oncogene.

[B6] Foekens JA, Atkins D, Zhang Y, Sweep FC, Harbeck N, Paradiso A, Cufer T, Sieuwerts AM, Talantov D, Span PN, Tjan-Heijnen VC, Zito AF, Specht K, Hoefler H, Golouh R, Schittulli F, Schmitt M, Beex LV, Klijn JG, Wang Y (2006). Multicenter validation of a gene expression-based prognostic signature in lymph node-negative primary breast cancer. J Clin Oncol.

[B7] Hu Z, Fan C, Oh DS, Marron JS, He X, Qaqish BF, Livasy C, Carey LA, Reynolds E, Dressler L, Nobel A, Parker J, Ewend MG, Sawyer LR, Wu J, Liu Y, Nanda R, Tretiakova M, Ruiz Orrico A, Dreher D, Palazzo JP, Perreard L, Nelson E, Mone M, Hansen H, Mullins M, Quackenbush JF, Ellis MJ, Olopade OI, Bernard PS, Perou CM (2006). The molecular portraits of breast tumors are conserved across microarray platforms. BMC Genomics.

[B8] Huang E, Ishida S, Pittman J, Dressman H, Bild A, Kloos M, D'Amico M, Pestell RG, West M, Nevins JR (2003). Gene expression phenotypic models that predict the activity of oncogenic pathways. Nat Genet.

[B9] Korkola JE, DeVries S, Fridlyand J, Hwang ES, Estep AL, Chen YY, Chew KL, Dairkee SH, Jensen RM, Waldman FM (2003). Differentiation of lobular versus ductal breast carcinomas by expression microarray analysis. Cancer Res.

[B10] Ma XJ, Wang Z, Ryan PD, Isakoff SJ, Barmettler A, Fuller A, Muir B, Mohapatra G, Salunga R, Tuggle JT, Tran Y, Tran D, Tassin A, Amon P, Wang W, Wang W, Enright E, Stecker K, Estepa-Sabal E, Smith B, Younger J, Balis U, Michaelson J, Bhan A, Habin K, Baer TM, Brugge J, Haber DA, Erlander MG, Sgroi DC (2004). A two-gene expression ratio predicts clinical outcome in breast cancer patients treated with tamoxifen. Cancer Cell.

[B11] Miller LD, Smeds J, George J, Vega VB, Vergara L, Ploner A, Pawitan Y, Hall P, Klaar S, Liu ET, Bergh J (2005). An expression signature for p53 status in human breast cancer predicts mutation status, transcriptional effects, and patient survival. Proc Natl Acad Sci USA.

[B12] Naderi A, Teschendorff AE, Barbosa-Morais NL, Pinder SE, Green AR, Powe DG, Robertson JF, Aparicio S, Ellis IO, Brenton JD, Caldas C (2007). A gene-expression signature to predict survival in breast cancer across independent data sets. Oncogene.

[B13] Pawitan Y, Bjohle J, Amler L, Borg AL, Egyhazi S, Hall P, Han X, Holmberg L, Huang F, Klaar S, Liu ET, Miller L, Nordgren H, Ploner A, Sandelin K, Shaw PM, Smeds J, Skoog L, Wedrén S, Bergh J (2005). Gene expression profiling spares early breast cancer patients from adjuvant therapy: derived and validated in two population-based cohorts. Breast Cancer Res.

[B14] Richardson AL, Wang ZC, De Nicolo A, Lu X, Brown M, Miron A, Liao X, Iglehart JD, Livingston DM, Ganesan S (2006). X chromosomal abnormalities in basal-like human breast cancer. Cancer Cell.

[B15] Sorlie T, Perou CM, Tibshirani R, Aas T, Geisler S, Johnsen H, Hastie T, Eisen MB, Rijn M van de, Jeffrey SS, Thorsen T, Quist H, Matese JC, Brown PO, Botstein D, Eystein Lønning P, Børresen-Dale AL (2001). Gene expression patterns of breast carcinomas distinguish tumor subclasses with clinical implications. Proc Natl Acad Sci USA.

[B16] Sorlie T, Tibshirani R, Parker J, Hastie T, Marron JS, Nobel A, Deng S, Johnsen H, Pesich R, Geisler S, Demeter J, Perou CM, Lønning PE, Brown PO, Børresen-Dale AL, Botstein D (2003). Repeated observation of breast tumor subtypes in independent gene expression data sets. Proc Natl Acad Sci USA.

[B17] Sotiriou C, Neo SY, McShane LM, Korn EL, Long PM, Jazaeri A, Martiat P, Fox SB, Harris AL, Liu ET (2003). Breast cancer classification and prognosis based on gene expression profiles from a population-based study. Proc Natl Acad Sci USA.

[B18] Sotiriou C, Wirapati P, Loi S, Harris A, Fox S, Smeds J, Nordgren H, Farmer P, Praz V, Haibe-Kains B, Desmedt C, Larsimont D, Cardoso F, Peterse H, Nuyten D, Buyse M, Vijver MJ Van de, Bergh J, Piccart M, Delorenzi M (2006). Gene expression profiling in breast cancer: understanding the molecular basis of histologic grade to improve prognosis. J Natl Cancer Inst.

[B19] van't Veer LJ, Dai H, Vijver MJ van de, He YD, Hart AA, Mao M, Peterse HL, Kooy K van der, Marton MJ, Witteveen AT, Schreiber GJ, Kerkhoven RM, Roberts C, Linsley PS, Bernards R, Friend SH (2002). Gene expression profiling predicts clinical outcome of breast cancer. Nature.

[B20] Vijver MJ van de, He YD, van't Veer LJ, Dai H, Hart AA, Voskuil DW, Schreiber GJ, Peterse JL, Roberts C, Marton MJ, Parrish M, Atsma D, Witteveen A, Glas A, Delahaye L, Velde T van der, Bartelink H, Rodenhuis S, Rutgers ET, Friend SH, Bernards R (2002). A gene-expression signature as a predictor of survival in breast cancer. N Engl J Med.

[B21] Wang Y, Klijn JG, Zhang Y, Sieuwerts AM, Look MP, Yang F, Talantov D, Timmermans M, Meijer-van Gelder ME, Yu J, Jatkoe T, Berns EM, Atkins D, Foekens JA (2005). Gene-expression profiles to predict distant metastasis of lymph-node-negative primary breast cancer. Lancet.

[B22] Paik S, Tang G, Shak S, Kim C, Baker J, Kim W, Cronin M, Baehner FL, Watson D, Bryant J, Costantino JP, Geyer CE, Wickerham DL, Wolmark N (2006). Gene expression and benefit of chemotherapy in women with node-negative, estrogen receptor-positive breast cancer. J Clin Oncol.

[B23] Chang HY, Nuyten DS, Sneddon JB, Hastie T, Tibshirani R, Sorlie T, Dai H, He YD, van't Veer LJ, Bartelink H, Rijn M van de, Brown PO, Vijver MJ van de (2005). Robustness, scalability, and integration of a wound-response gene expression signature in predicting breast cancer survival. Proc Natl Acad Sci USA.

[B24] Chang HY, Sneddon JB, Alizadeh AA, Sood R, West RB, Montgomery K, Chi JT, Rijn M van de, Botstein D, Brown PO (2004). Gene expression signature of fibroblast serum response predicts human cancer progression: similarities between tumors and wounds. PLoS Biol.

[B25] Fan C, Oh DS, Wessels L, Weigelt B, Nuyten DS, Nobel AB, van't Veer LJ, Perou CM (2006). Concordance among gene-expression-based predictors for breast cancer. N Engl J Med.

[B26] Maglott D, Ostell J, Pruitt KD, Tatusova T (2007). Entrez Gene: gene-centered information at NCBI. Nucleic Acids Res.

[B27] MAQC Consortium, Shi L, Reid LH, Jones WD, Shippy R, Warrington JA, Baker SC, Collins PJ, de Longueville F, Kawasaki ES, Lee KY, Luo Y, Sun YA, Willey JC, Setterquist RA, Fischer GM, Tong W, Dragan YP, Dix DJ, Frueh FW, Goodsaid FM, Herman D, Jensen RV, Johnson CD, Lobenhofer EK, Puri RK, Schrf U, Thierry-Mieg J, Wang C, Wilson M (2006). The MicroArray Quality Control (MAQC) project shows inter- and intraplatform reproducibility of gene expression measurements. Nat Biotechnol.

[B28] Hedges LV, Olkin I (1985). Statistical Methods for Meta-Analysis.

[B29] McLachlan G, Peel D (2000). Finite Mixture Models.

[B30] Therneau TM (1999). A Package for Survival Analysis in S.

[B31] Sum EY, Peng B, Yu X, Chen J, Byrne J, Lindeman GJ, Visvader JE (2002). The LIM domain protein LMO4 interacts with the cofactor CtIP and the tumor suppressor BRCA1 and inhibits BRCA1 activity. J Biol Chem.

[B32] Ivshina AV, George J, Senko O, Mow B, Putti TC, Smeds J, Lindahl T, Pawitan Y, Hall P, Nordgren H, Wong JE, Liu ET, Bergh J, Kuznetsov VA, Miller LD (2006). Genetic reclassification of histologic grade delineates new clinical subtypes of breast cancer. Cancer Res.

[B33] Perou CM, Sorlie T, Eisen MB, Rijn M van de, Jeffrey SS, Rees CA, Pollack JR, Ross DT, Johnsen H, Akslen LA, Fluge O, Pergamenschikov A, Williams C, Zhu SX, Lønning PE, Børresen-Dale AL, Brown PO, Botstein D (2000). Molecular portraits of human breast tumours. Nature.

[B34] Bogaerts J, Cardoso F, Buyse M, Braga S, Loi S, Harrison JA, Bines J, Mook S, Decker N, Ravdin P, Therasse P, Rutgers E, van't Veer LJ, Piccart M, TRANSBIG consortium (2006). Gene signature evaluation as a prognostic tool: challenges in the design of the MINDACT trial. Nat Clin Pract Oncol.

[B35] Sparano JA (2006). TAILORx: trial assigning individualized options for treatment (Rx). Clin Breast Cancer.

[B36] Teschendorff AE, Miremadi A, Pinder SE, Ellis IO, Caldas C (2007). An immune response gene expression module identifies a good prognosis subtype in estrogen receptor negative breast cancer. Genome Biol.

[B37] Alexe G, Dalgin GS, Scanfeld D, Tamayo P, Mesirov JP, DeLisi C, Harris L, Barnard N, Martel M, Levine AJ, Ganesan S, Bhanot G (2007). High expression of lymphocyte-associated genes in node-negative HER2^+ ^breast cancers correlates with lower recurrence rates. Cancer Res.

[B38] Blamey RW (1996). The design and clinical use of the Nottingham Prognostic Index in breast cancer. The Breast.

[B39] (1998). Tamoxifen for early breast cancer: an overview of the randomised trials. Early Breast Cancer Trialists' Collaborative Group. Lancet.

[B40] Romond EH, Perez EA, Bryant J, Suman VJ, Geyer CE, Davidson NE, Tan-Chiu E, Martino S, Paik S, Kaufman PA, Swain SM, Pisansky TM, Fehrenbacher L, Kutteh LA, Vogel VG, Visscher DW, Yothers G, Jenkins RB, Brown AM, Dakhil SR, Mamounas EP, Lingle WL, Klein PM, Ingle JN, Wolmark N (2005). Trastuzumab plus adjuvant chemotherapy for operable HER2-positive breast cancer. N Engl J Med.

[B41] International Genomics Consortium. http://www.intgen.org.

[B42] Paik S, Shak S, Tang G, Kim C, Baker J, Cronin M, Baehner FL, Walker MG, Watson D, Park T, Hiller W, Fisher ER, Wickerham DL, Bryant J, Wolmark N (2004). A multigene assay to predict recurrence of tamoxifen-treated, node-negative breast cancer. N Engl J Med.

[B43] Teschendorff AE, Naderi A, Barbosa-Morais NL, Pinder SE, Ellis IO, Aparicio S, Brenton JD, Caldas C (2006). A consensus prognostic gene expression classifier for ER positive breast cancer. Genome Biol.

[B44] Whitfield ML, George LK, Grant GD, Perou CM (2006). Common markers of proliferation. Nat Rev Cancer.

