# Supplementary material for: Meta-analysis of gene expression profiles in breast cancer: toward a unified understanding of breast cancer subtyping and prognosis signatures
Source: Breast Cancer Res. 2008 Jul 28;10(4):R65. doi: 10.1186/bcr2124 (PMC2575538; doi:10.1186/bcr2124)
Supplement: Additional file 1 — Supplementary methods. Supplementary methods including the following sections: 'Probe annotation and gene matching', 'Preprocessing of expression values', 'Identifying coexpression modules', 'Module scores', 'Clustering and multimodality tests', 'Survival analysis', 'Cell-cycle periodicity' and 'Cross-platform applications of signatures'. [file bcr2124-S1.pdf]

Supplementary Methods

Probe annotation and gene matching

Hybridization probes were mapped to Entrez GeneID(Maglott *et al.*, 2005) through sequence alignment against RefSeq mRNA in the well-curated (NM) subset, similar to the approach by Shi *et al.*(Shi *et al.*, 2006). Affymetrix and Agilent probe sequences were obtained from the manufacturer. For other platforms (cDNA or Agilent with unavailable probe sequences), the mapping was done by retrieving the GenBank sequences corresponding to the probes. Alignment is done using BLAT(Kent, 2002). Alternate transcripts (different RefSeq with the same GeneID) were considered to be the same "gene". RefSeq version 21 (2007.01.21) and Entrez database version 2007.01.21 were used. When multiple probes were mapped to the same GeneID, the one with the highest variance in a particular dataset was selected to represent the GeneID.

Preprocessing of expression values

We used the normalized expression measures ($\log_2$ of intensity in single-channel platforms or $\log_2$ratio in dual-channel platforms) published by the original studies. Because our meta-analytical approach combines summary statistics instead of expression data, normalization across datasets was not necessary. Within each dataset, missing values were imputed using the mean of present values for the same gene.

Identifying coexpression modules

The expression levels of the prototype genes on the $\log_2$ scale were used as explanatory variables in multiple regression with Gaussian error model, using the following equation (gene symbols stand for their log expression and coefficients are omitted for clarity):

$$Y_i = \text{ESR1} + \text{ERBB2} + \text{AURKA}$$

where the response variable Y_i is the expression of gene i. This model is fitted separately for each gene i in the array. The association between gene i and prototype j (in the presence of or conditional on all other prototypes) is tested using the t-statistic for each coefficient. Because the t-statistics for different datasets have different degrees of freedom, we put them all on the same scale by transforming to the corresponding cumulative probabilities and then to Z-scores using the inverse standard normal cumulative distribution function.

The linear model above was fitted separately to each gene in each dataset, and the Z-scores were combined meta-analytically over K studies using the

"inverse normal method"(Hedges and Olkin, 1985):

$$\overline{Z}_{ij} = \sum_{k=1}^{K} Z_{ijk} / \sqrt{K_i}.$$

where i, j and k are indices for genes, tested regression terms and dataset, respectively. K_i is the number of datasets where the gene i is present (that is, any platform missing the gene is ignored). A schematic is shown in figure 1 of this document.

Due to the large sample size, the conservative Bonferroni-corrected p-value of less than 0.05 is achieved for many coefficients (for a test of any departure from linear independence, $\beta_{ij} = 0$). To select genes that are most strongly associated with the prototypes, we use a more stringent criterion of $|Z| \geq 16$ instead, which is well above $|Z| \approx 5$, that corresponds to a corrected p-value of 0.05.

Some genes have more than one prototype satisfying the criteria above. To make the genes in a module more specific, we introduced a "uniqueness" criterion:

$$U_{ij} = Z_{ij}^2 / \sum_q Z_{iq}^2$$

where q is the index over all prototypes. Thus a gene i is a part of module j when $Z_{ij} \geq 16$ and $U_{ij} > 0.5$.

Module scores

For a specific dataset, the module score is computed for each sample as:

$$\text{module score} = \sum_i w_i x_i / \sum_i |w_i|$$

where x_i is the expression of a gene in the module that is present in the dataset's platform. w_i is either $+1$ or -1 depending on the sign of the Z-score of the association with the prototypes. The denominator is used so that the range of the module score roughly corresponds to the typical range of log expression values. Mean centering is performed on each gene prior to module score calculation, to make the score roughly centered around zero. However, these scaling and centering are only for convenience in displaying the scores. Our analyses do not assume commensurability of module scores between datasets.

Clustering and multimodality tests

Gaussian mixture models(McLachlan and Peel, 2000) with equal variance for all clusters were fitted. In the case of two-dimensional data, diagonal covariance matrices were used, allowing for dimension-specific variances. For testing multimodality, we used the likelihood ratio test statistics between the fitted model for the tested number of components, k, versus the simpler model with $k - 1$ components. The null distribution was generated by parametric bootstrapping from the fitted model with $k - 1$ components.

Each tumor was automatically classified as type 1, 2 or 3 using the posterior probability of membership in the clusters. Although there were a few ambiguous

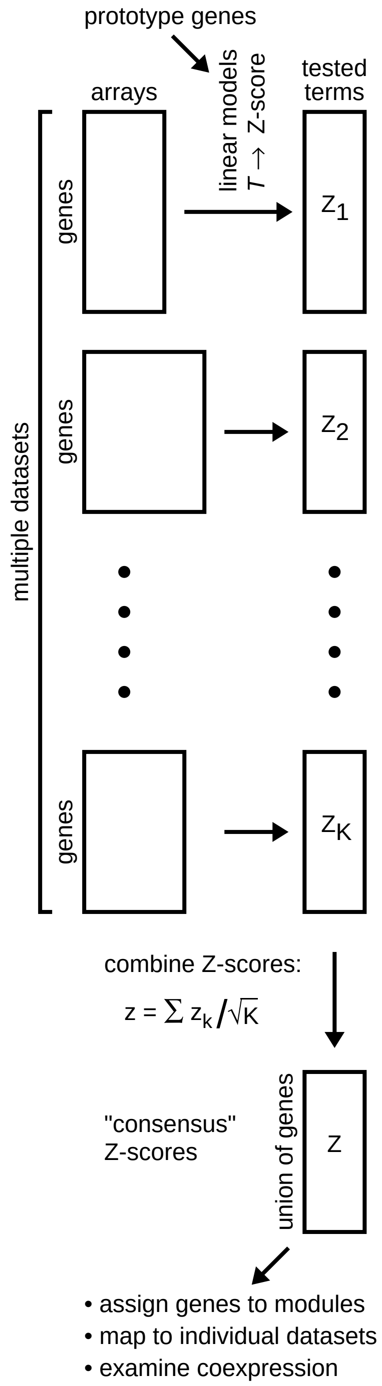

Figure 1: General flowchart for meta-analysis of multi-platform gene expression datasets.

cases with low probabilities shared among clusters (such as points in between clusters in Figure 3b), for simplicity all tumors were strictly categorized into either one type using the criterion of maximum probability.

Survival analysis

Detailed treatment of survival endpoints and time units can be found in **Supplementary Result 1.1**. Because not all studies reported the complete data on all endpoints, we only showed results for one or combined endpoints. Figure 5 and 6 were based on metastasis-free survival (if available) or overall survival. Complete results for each type of endpoints can be found in **Supplementary Result 1, 5-8**.

Survival curves and 5-year survivals in forest plots were based on Kaplan-Meier estimates, with the Greenwood method for computing the 95% confidence intervals(Therneau, 1999). Hazard ratios between two groups were calculated using Cox regression. Stratified Cox regression was used to compute total hazard ratios in forest plots and multivariate analysis in Figure 4f, using the dataset as the stratum indicator, thus allowing for different baseline hazard functions between cohorts.

In multivariate analysis, tumor size is categorized as small or large using the 2-cm cutoff because some datasets provide only the category instead of the continuous length measurements. Histological grade is dichotomized into grade 1 versus 2+3 because it is the most clinically relevant for identifying the low-risk subset (contrasting 1+2 versus 3 will give, respectively, intermediate and high risk groups not appropriate for chemotherapy decision).

Cox regression was used to compute gene-by-gene Z-survival scores, treating the log expression measures as continuous explanatory variables. The Z-score was based on the signed square-root of the deviance (two times the log likelihood ratio). These Z-scores were combined across datasets using the same meta-analytical formula as used for coexpression module analysis, described above.

Cell-cycle periodicity

We used datasets from various cell cycle experiments reported in Whitfield *et al.*(Whitfield *et al.*, 2002). The periodicity of expression was scored using linear models with a pair of cosine and sine functions as the explanatory variable, with the frequency corresponding to the estimated cell-cycle periodicities in the respective experiments. The F-ratio test statistics were converted to p-values with the appropriate degrees of freedom, and then converted to Z-scores using the inverse standard normal distribution function. The Z-scores from different experiments were combined meta-analytically as described above.

Cross-platform applications of signatures

Only genes in the signatures that can be mapped to GeneID were used. A prediction score was computed for each signature, using a linear combination similar to the formula for module score above. Gene-specific weights (coefficients, correlations, or other measures) from the original studies were used (or, if not available, +1 or -1 depending on the original up- or down-regulation of each gene). Genes absent in a platform were ignored. The scales of the prediction scores were not comparable between signatures. For each cohort and

signature combination, a cutoff was chosen based on the percentile of the scores (33% for the results in Figure 6).

The signature EMC-76 had different gene lists for ER- and ER+ tumors, and were applied accordingly (except in proliferation-only partial signatures that were applied to the all patients regardless of the ER status because the ER- signature does not contain proliferation genes).

The signature ZCOX was identified from the data collection by selecting the top ranking genes according to survival association. The criterion was an absolute value of Z-score greater than 6 (shown in Figure 5b of the main text). To avoid bias in the performance assessment, we used a "leave-one-dataset-out" cross-validation: the test dataset (whose performance was reported) was excluded from the calculation of the meta-analytical Z-scores for selecting the tested signature.

References

Hedges L.V. and Olkin I. (1985). *Statistical methods for meta-analysis*. Academic Press, London.

Kent W.J. (2002). BLAT—the BLAST-like alignment tool. *Genome Res.*, **12**, 656–664.

Maglott D., Ostell J., Pruitt K.D. and Tatusova T. (2005). Entrez Gene: gene-centered information at NCBI. *Nucleic Acid Res.*, **33**, D53–D58.

McLachlan G. and Peel D. (2000). *Finite mixture models*. Wiley, New York.

Shi L., Reid L.H., Jones W.D., Shippy R., Warrington J.A., Baker S.C., Collins P.J., de Longueville F., Kawasaki E.S., Lee K.Y., Luo Y., Sun Y.A., Willey J.M., Setterquist R.A., Fischer G.M., Tong W., Dragan Y.P., Dix D.J., Frueh F.W., Goodsaid F.M., Herman D., Jensen R.V., Johnson C.D., Lobenhofer E.K., Puri R.K., Schrf U., Thierry-Mieg J., Wang C., Wilson M., Wolber P.K., Zhang L., Slikker W.J., Shi L. and Reid L.H. (2006). The MicroArray Quality Control (MAQC) project shows inter- and intraplatform reproducibility of gene expression measurements. *Nat Biotechnol*, **24**, 1151–1161.

Therneau T.M. (1999). A package for survival analysis in S. Tech. rep., Mayo Foundation.

Whitfield M.L., Sherlock G., Saldanha A.J., Murray J.I., Ball C.A., Alexander K.E., Matese J.C., Perou C.M., Hurt M.M., Brown P.O. and Botstein D. (2002). Identification of genes periodically expressed in the human cell cycle and their expression in tumors. *Mol. Biol. Cell*, **13**, 1977–2000.
