# Supplementary material for: Meta-analysis of gene expression profiles in breast cancer: toward a unified understanding of breast cancer subtyping and prognosis signatures
Source: Breast Cancer Res. 2008 Jul 28;10(4):R65. doi: 10.1186/bcr2124 (PMC2575538; doi:10.1186/bcr2124)

Supplementary Results

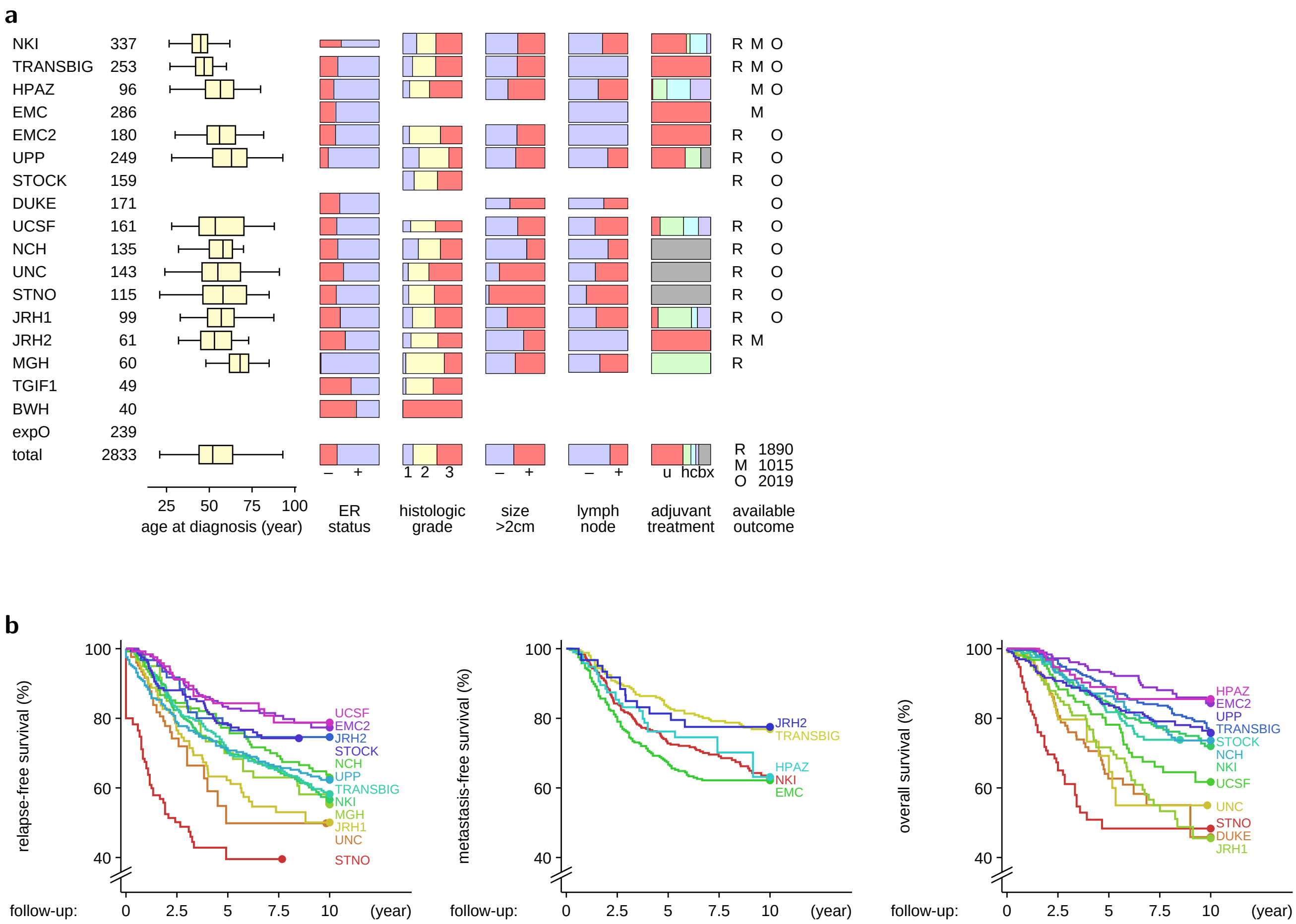

follow-up:	0	2.5	5	7.5	10	(year)
group:	number at risk:					events
STNO	115	39	10	1		60
UNC	128	33	10	4		32
JRH1	99	75	59	30	1	45
MGH	60	50	42	28	18	25
NCH	135	110	97	81	66	47
NKI	319	260	216	131	75	121
TRANSBIG	253	207	170	147	118	101
UPP	249	185	158	140	107	88
JRH2	61	55	44	38	33	15
STOCK	159	140	124	68		40
EMC2	180	164	149	94	40	37
UCSF	132	97	68	37	11	20
total	1890	1415	1147	799	469	631

follow-up:	0	2.5	5	7.5	10	(year)
group:	number at risk:					events
NKI	319	266	223	135	82	105
TRANSBIG	253	225	197	177	149	56
EMC	286	226	180	127	44	107
HPAZ	96	80	49	16	2	25
JRH2	61	55	44	36	31	13
total	1015	852	693	491	308	306

follow-up:	0	2.5	5	7.5	10	(year)
group:	number at risk:					events
STNO	115	54	14	3	1	46
DUKE	170	86	46	12	3	43
UNC	129	39	14	5		22
JRH1	99	85	70	32	1	45
UCSF	132	104	74	43	14	37
NKI	319	290	248	147	89	74
STOCK	159	148	130	64		40
NCH	135	122	111	96	81	34
TRANSBIG	253	240	212	190	154	57
UPP	232	198	173	152	122	51
EMC2	180	175	166	103	44	23
HPAZ	96	87	55	20	3	12
total	2019	1628	1313	867	512	484

Figure 1: Summaries of patient characteristics for each dataset. a) Distribution of important clinical variables, shown by boxplots for continuous variables and by colored bars showing proportions for categorical variables. The heights of the bars correspond to proportion of non-missing values. For adjuvant treatment, u = untreated, h = hormone therapy, c = chemotherapy, b = both, x = unspecified. For 'available outcome', the reported endpoints are: R = any relapse (unspecified), M = distant metastasis and O = overall survival. The numbers of patients with available follow-up data for each type of outcome are shown on the "total" line. b) Kaplan-Meier curves showing the heterogeneity of survival when the patients were stratified by the cohorts.

H

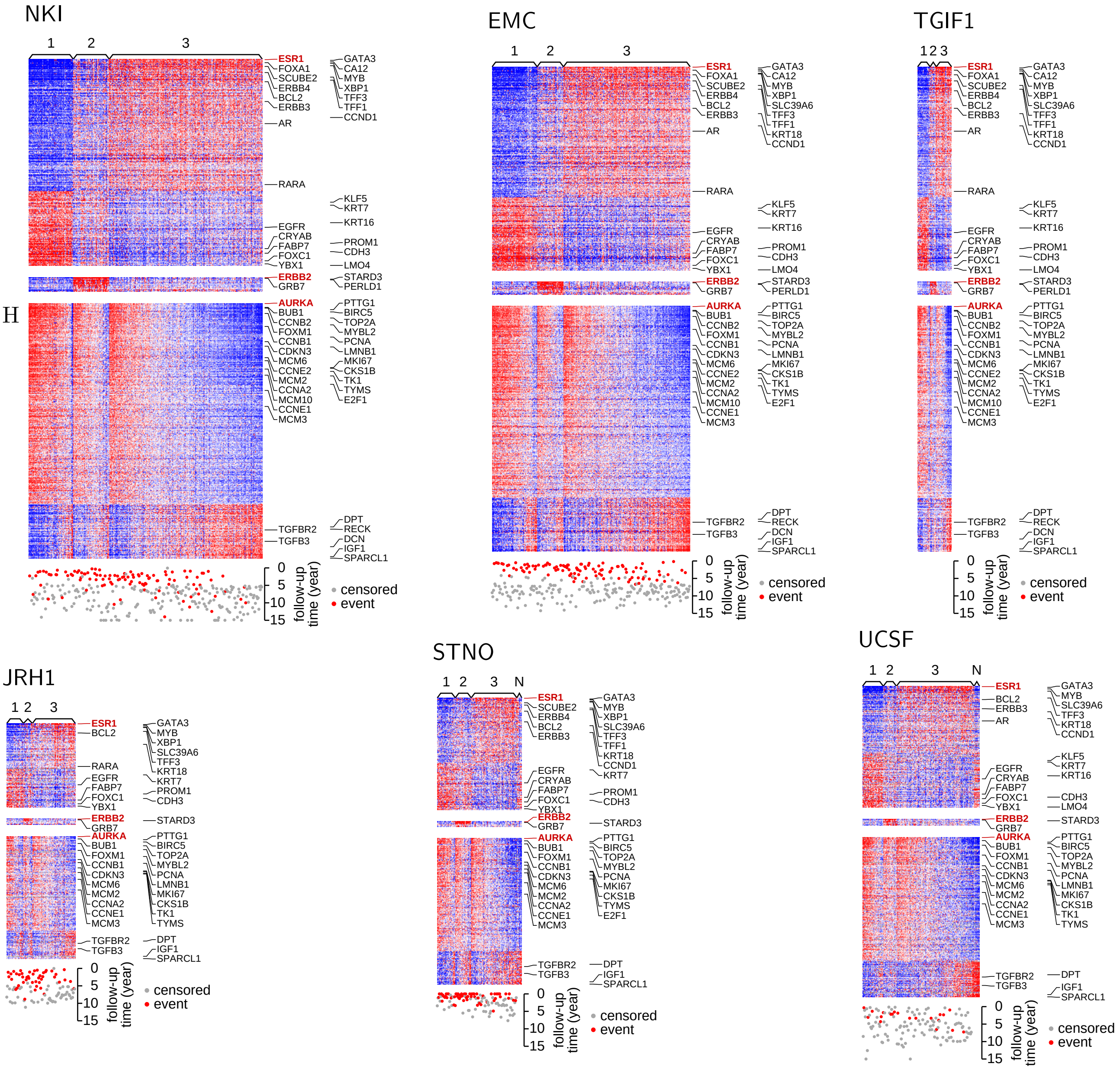

Figure 2: Heatmaps demonstrating the coexpression modules for all datasets (figures continued on the following two pages). The rows are genes, grouped according to the modules, whose gene symbol of the prototypes are shown in red. Within each group, the genes are ranked according to their Z-scores of association with their respective prototype. The columns are tumors, grouped according to subtype 1 (ER-/HER2-), 2 (HER2+), or 3 (ER+/HER2-). Within each subtype the tumors are sorted according to the average expression of the proliferation (AURKA) module. Genes within a module show strong correlated or anticorrelated expression with the prototype gene. The names of several well-known genes are shown to the right of each module. Underneath the heatmaps are patient's survival data.

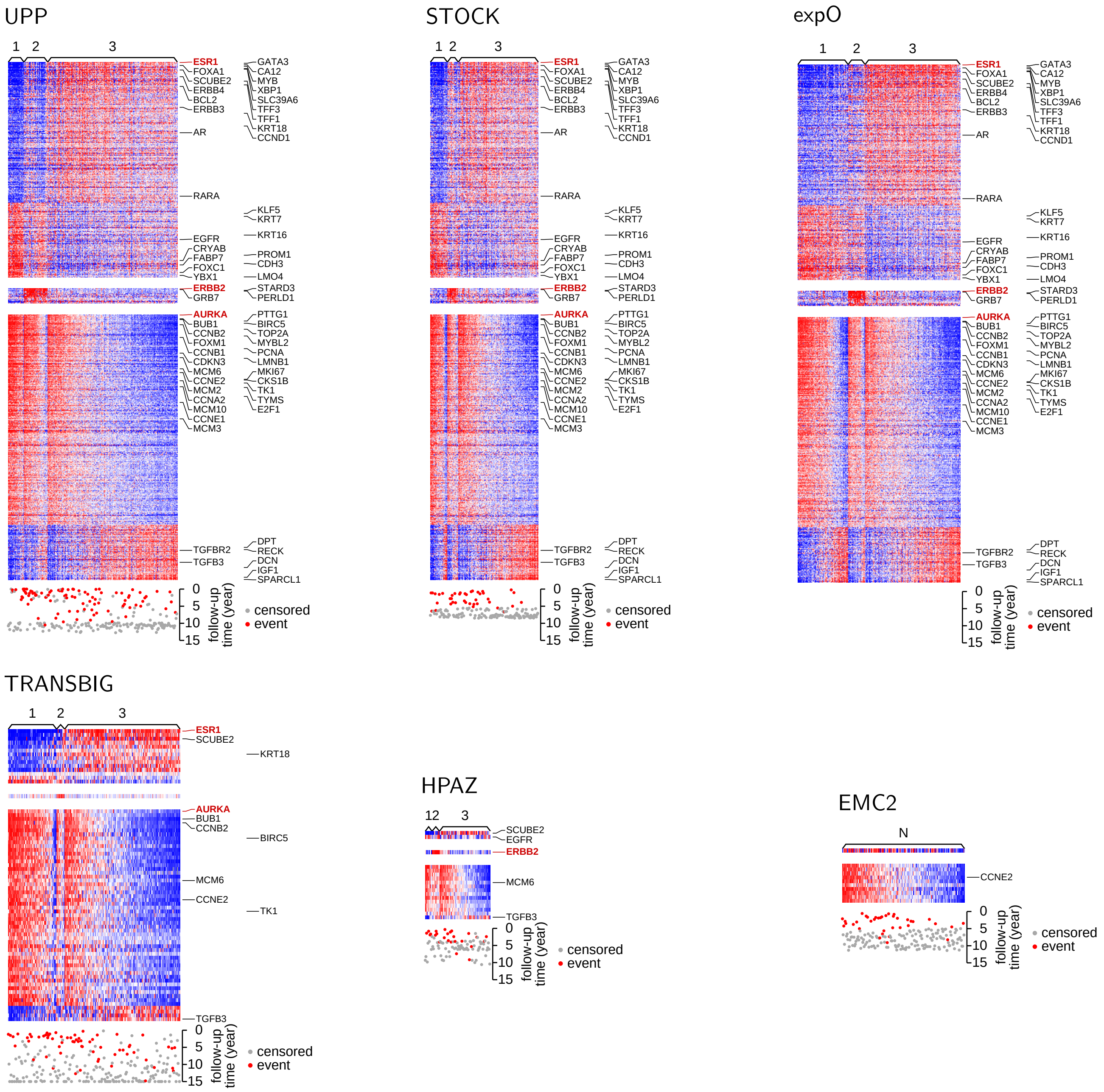

Note Because of the small number of genes mapped, the vertical scale of the heatmaps for TRANSBIG, HPAZ and EMC2 is larger than that of other datasets.

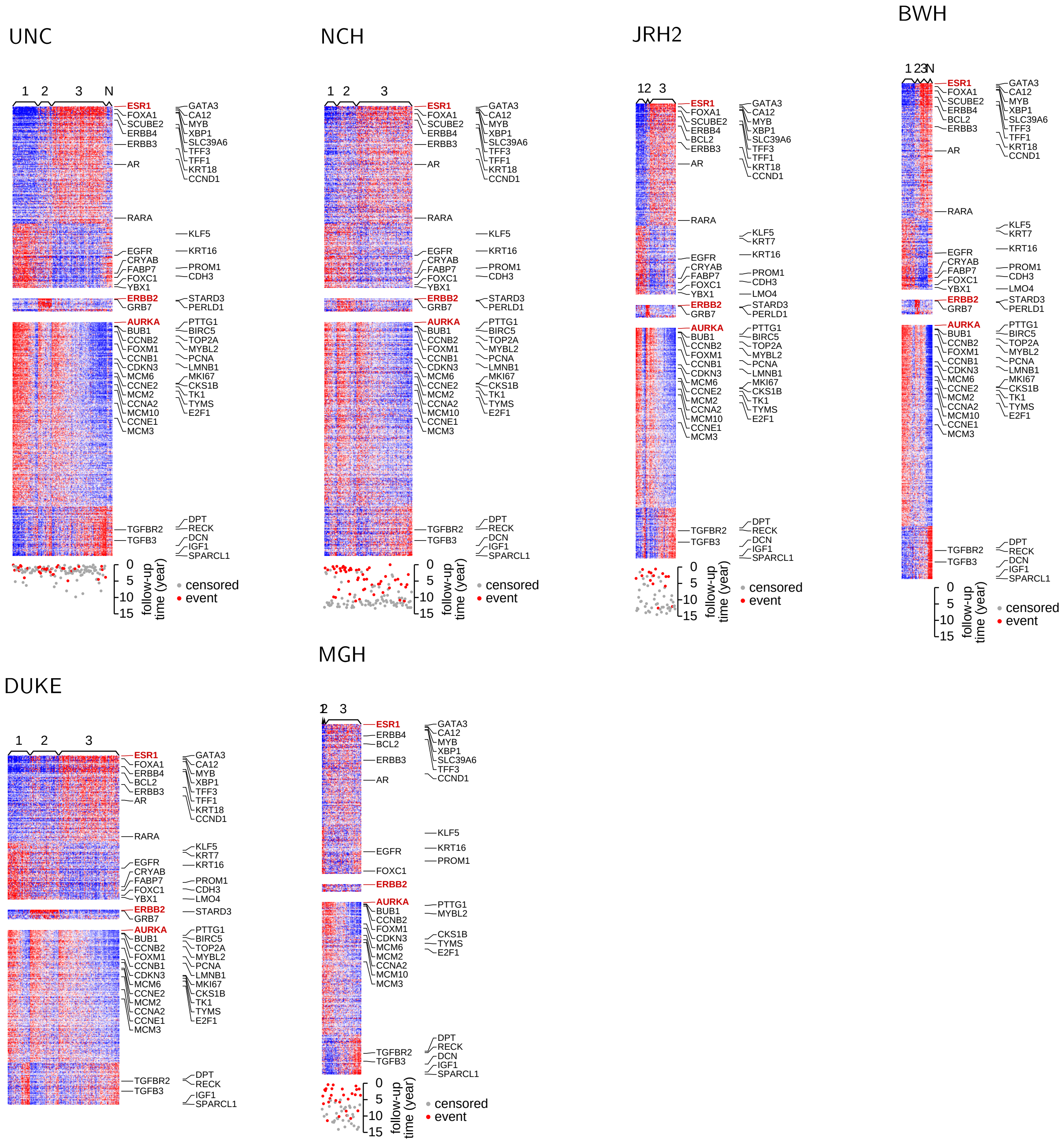

UNC
1 2 3 N
ESR1
FOXA1
SCUBE2
ERBB4
ERBB3
AR
RARA
EGFR
CRYAB
FABP7
FOXC1
YBX1
ERBB2
GRB7
AURKA
BUB1
CCNB2
FOXM1
CCNB1
CDKN3
MCM6
CCNE2
MCM2
CCNA2
MCM10
CCNE1
MCM3
TGFBR2
TGFB3
GATA3
CA12
MYB
XBP1
SLC39A6
TFF3
TFF1
KRT18
CCND1
KLF5
KRT16
PROM1
CDH3
STARD3
PERLD1
PTTG1
BIRC5
TOP2A
MYBL2
PCNA
LMNB1
MKI67
CKS1B
TK1
TYMS
E2F1
DPT
RECK
DCN
IGF1
SPARCL1
0
5
10
15
follow-up time (year)
censored
event
NCH
1 2 3
JRH2
1 2 3
KRT7
LMO4
BWH
1 2 3 N
BCL2
DUKE
1 2 3
MGH
1 2 3

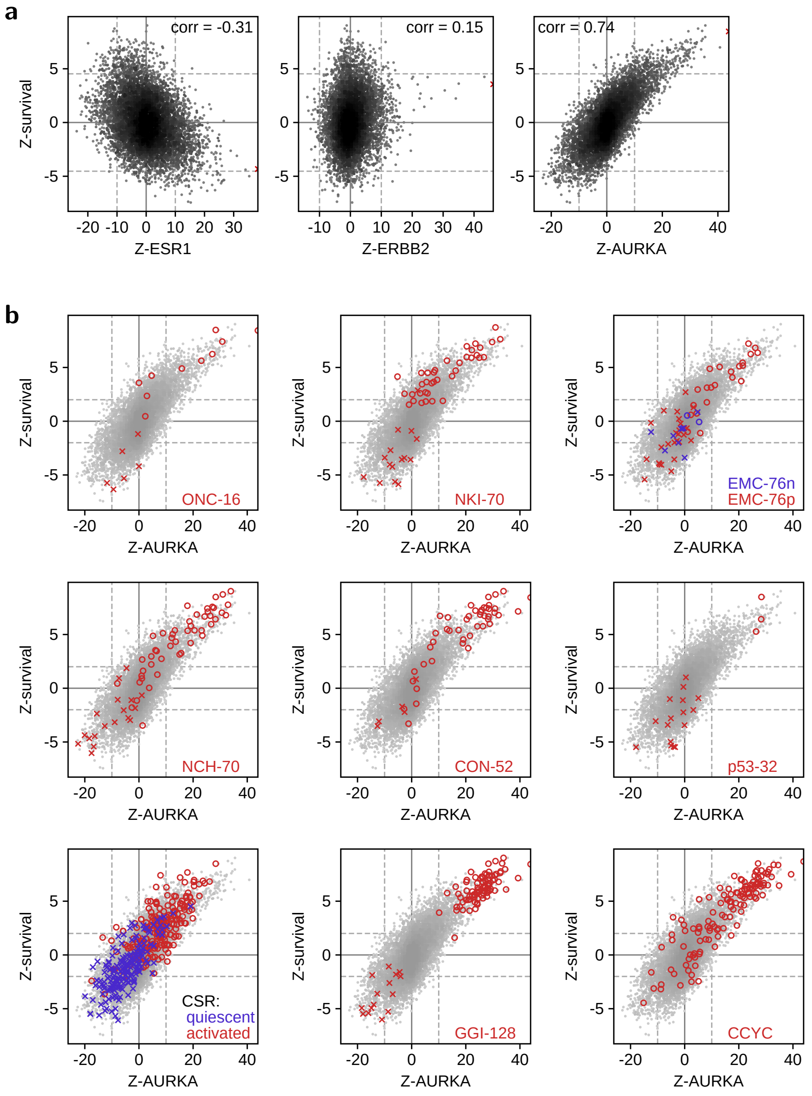

Figure 3: Prognostic power and modular association of individual genes. a) In each plot, the Z-score of survival association (vertical axis) is plotted against the Z-score of coexpression with a module prototype (horizontal axis) with their Pearson correlation coefficient indicated by "corr". The Z-survival of the prototype gene is shown by the cross on the right border. The band of (± 4.5) for Z-survival corresponds to Bonferroni correction for selecting from 17198 genes (at $p = 0.05$). The more stringent band of ± 10 is used for the Z-scores of coexpression. Strongly prognostic genes tend to be coexpressed with AURKA. b) Genes from various signatures (see table 2 in the main text) are overlaid on the scatter plots of Z-survival versus Z-AURKA. Circles and crosses respectively indicate positive and negative effects in the original studies. For signature EMC-76, the ER-positive and ER-negative signatures are denoted as EMC-76p and EMC-76n, respectively. The band for Z-survival (± 2) is the single-test significance level at $p = 0.05$ (more appropriate if the genes are already known). Many, but not all, signature genes are confirmed to be prognostic in the dataset collection.

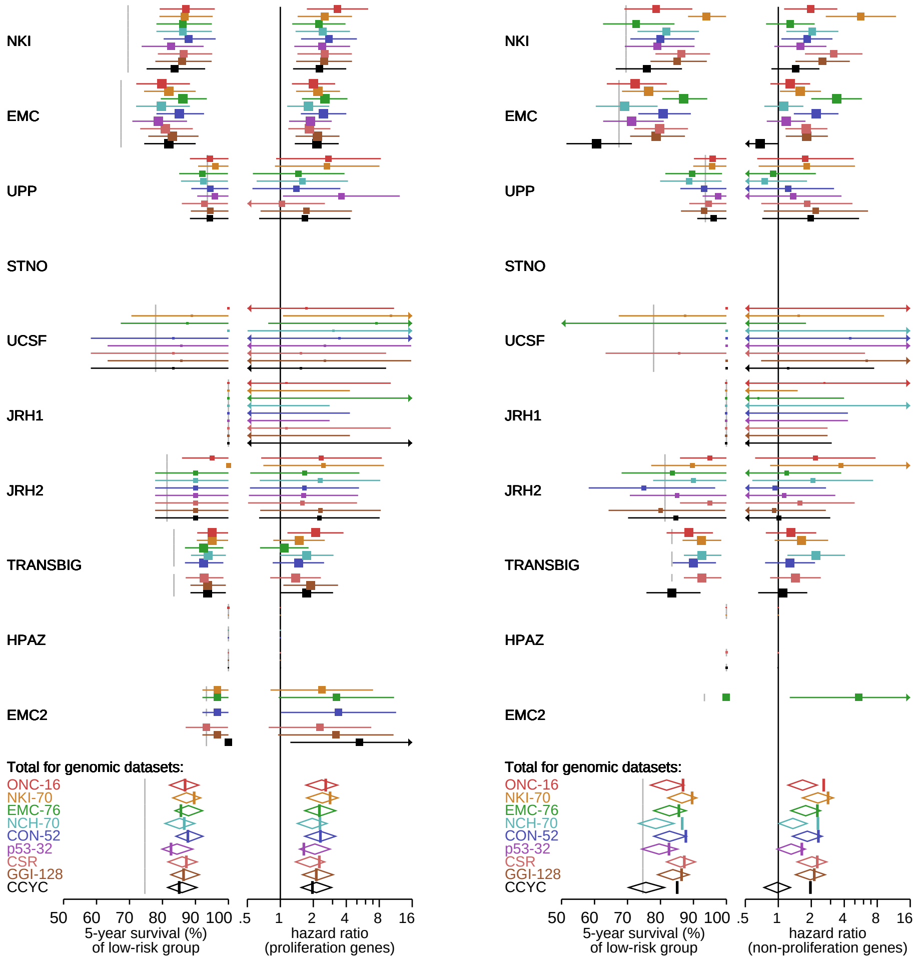

Figure 4: Detailed analysis of signature comparison. Per-dataset results corresponding to Figure 3 of the main text. The hazard ratios are stratified by datasets. Additionally, the Kaplan-Meier estimate of distant-relapse free survival at five-years for the low-risk group are shown (the estimates for high-risk group are not shown to avoid clutter). The vertical gray lines correspond to 5-year survival of the entire datasets (unstratified by risk groups). The two panels above correspond to the proliferation and non-proliferation gene subsets of untreated patients. Those for treated patients are shown on the following page. The dataset done on non-genomic platforms (TRANSBIG, HPAZ, and EMC2) were not considered when calculating the totals, due to lack of mapping for some signatures.

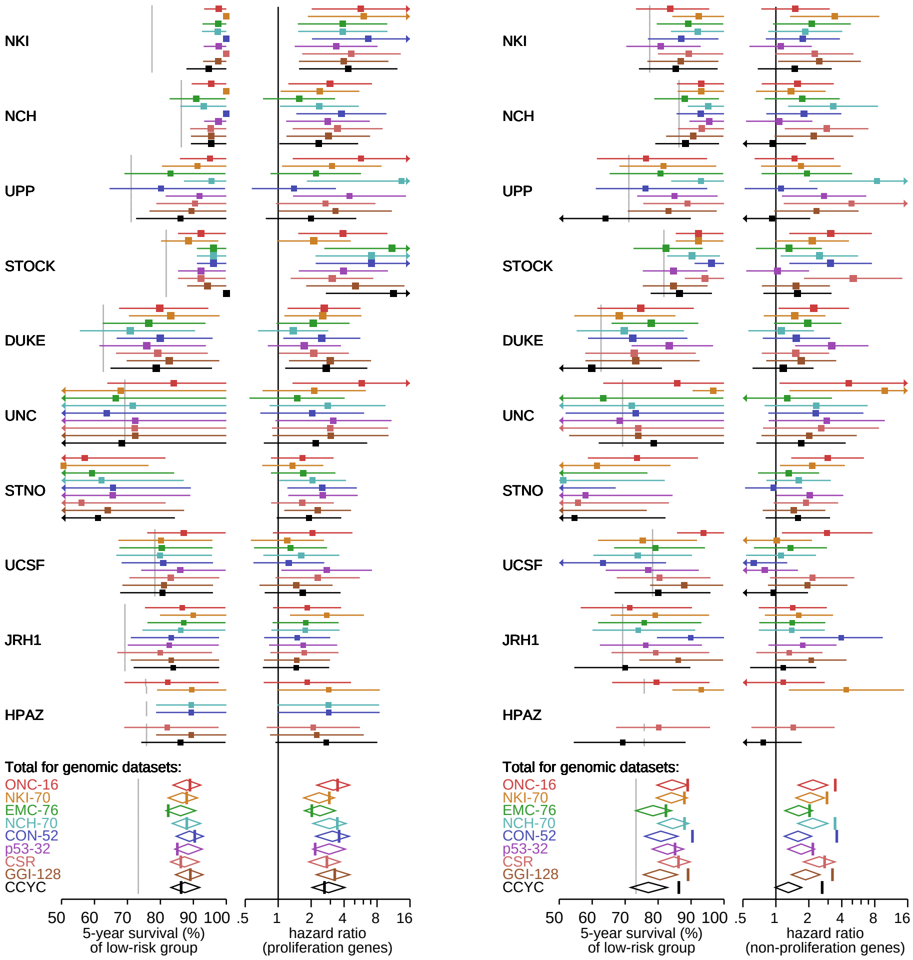

Figure 4 (continued). Results for systematically treated patients. See previous page for description.

Figure 5. Patient classifications made by different signatures (rows) when they are applied to various datasets (columns). This is the complete analysis, from which a few examples are shown in Figure 4 of the main text. The rows are signatures and the columns are datasets. All vertical axes correspond to proliferation score.

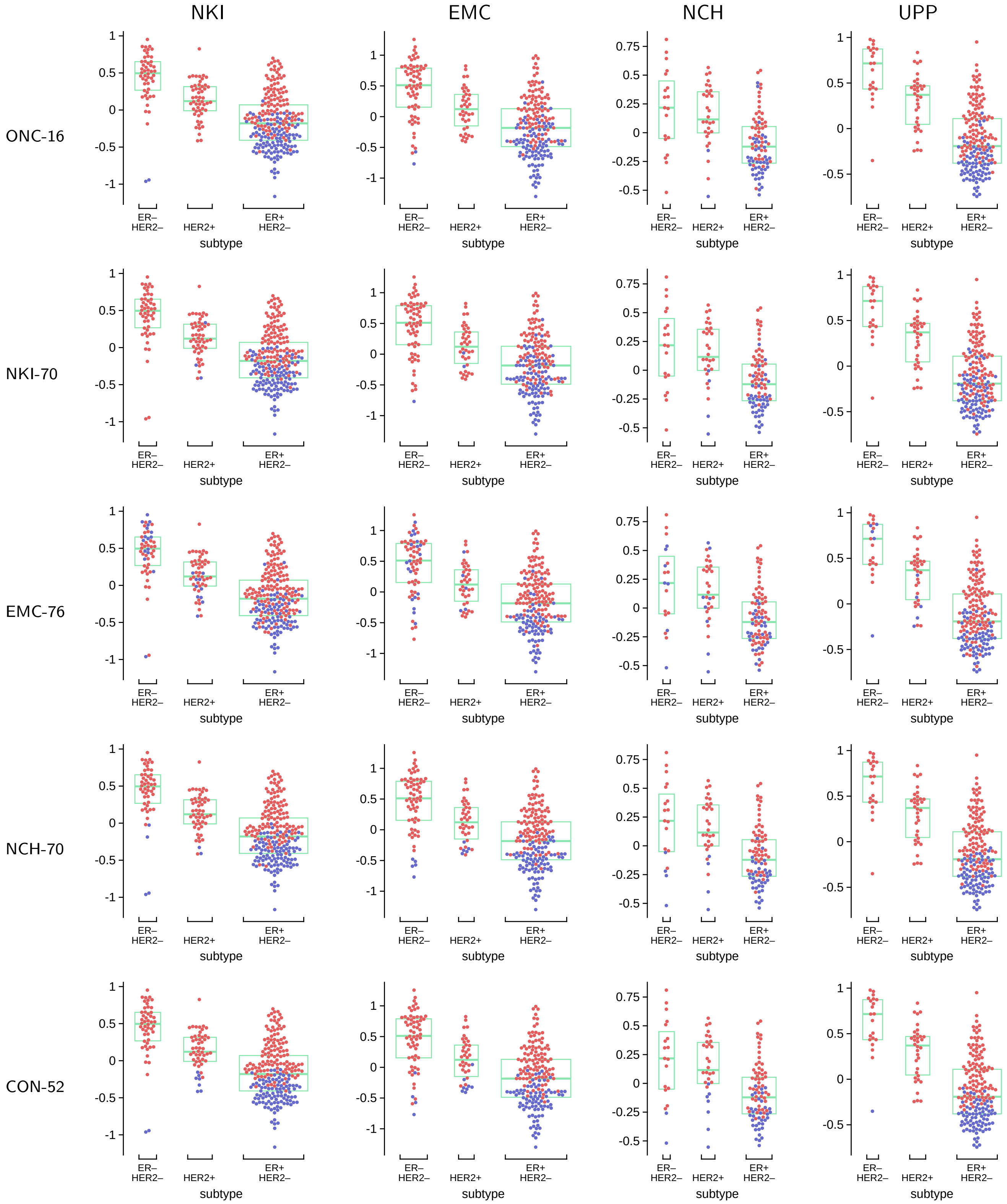

All vertical axes correspond to proliferation score.

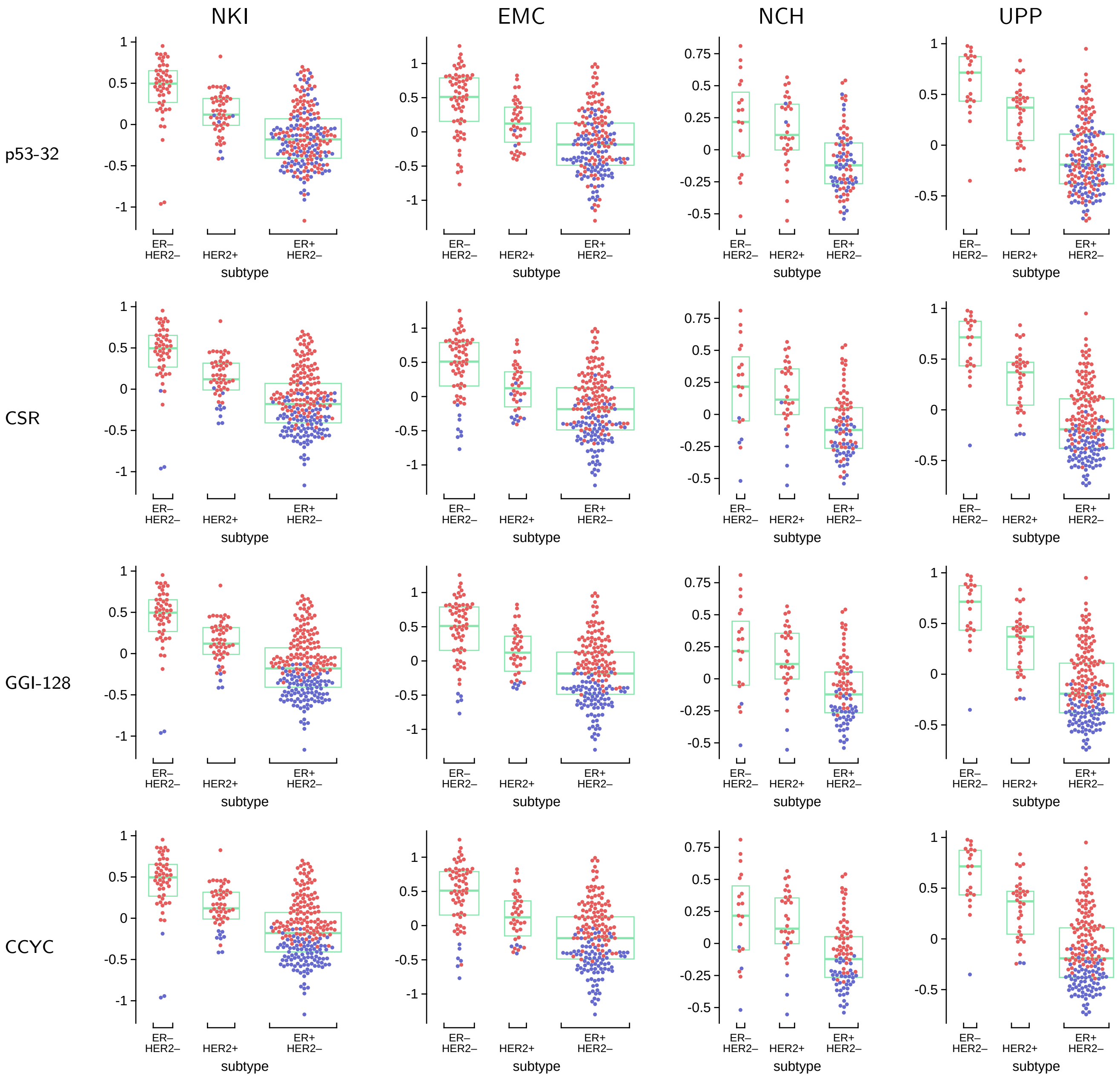

All vertical axes correspond to proliferation score.

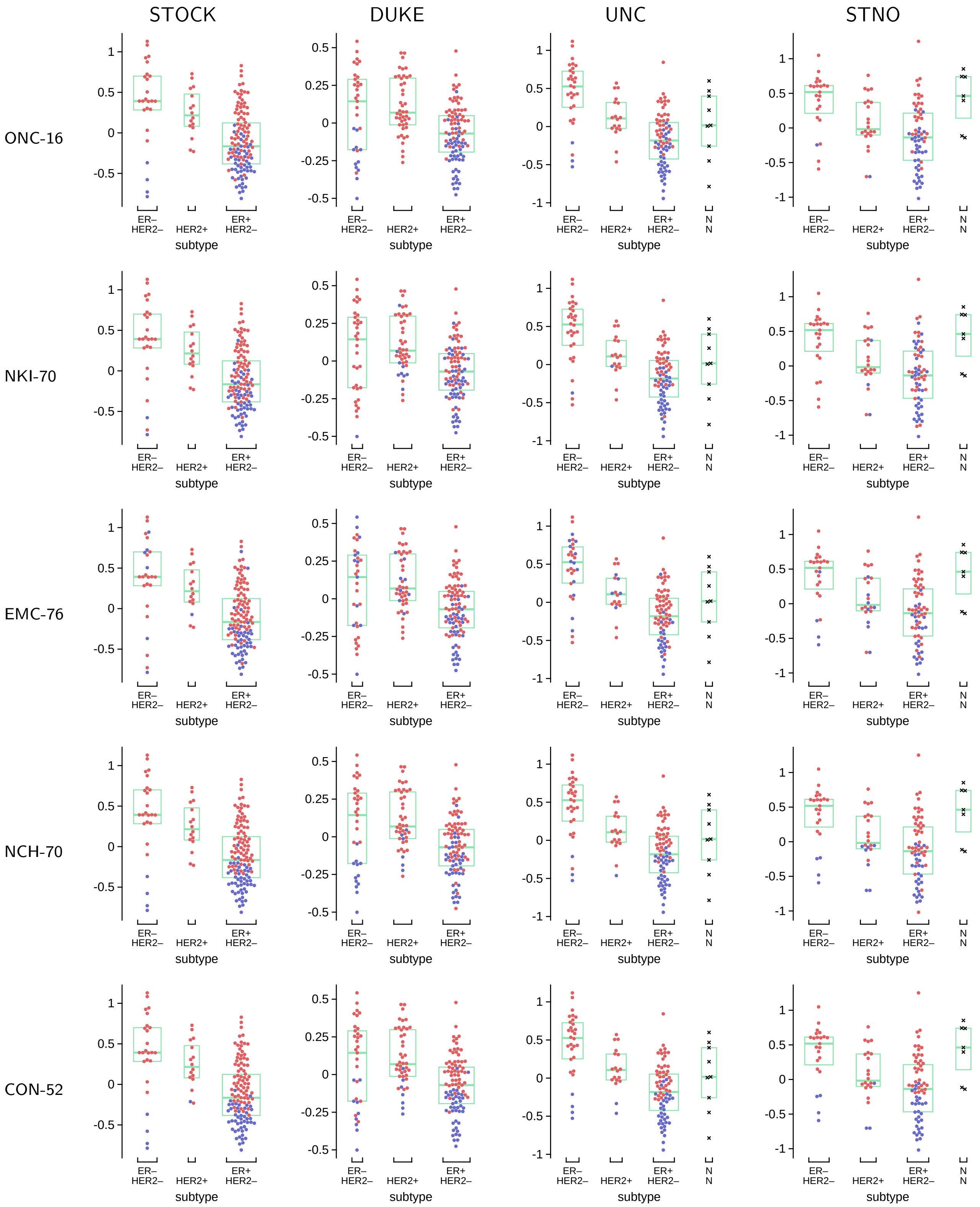

All vertical axes correspond to proliferation score.

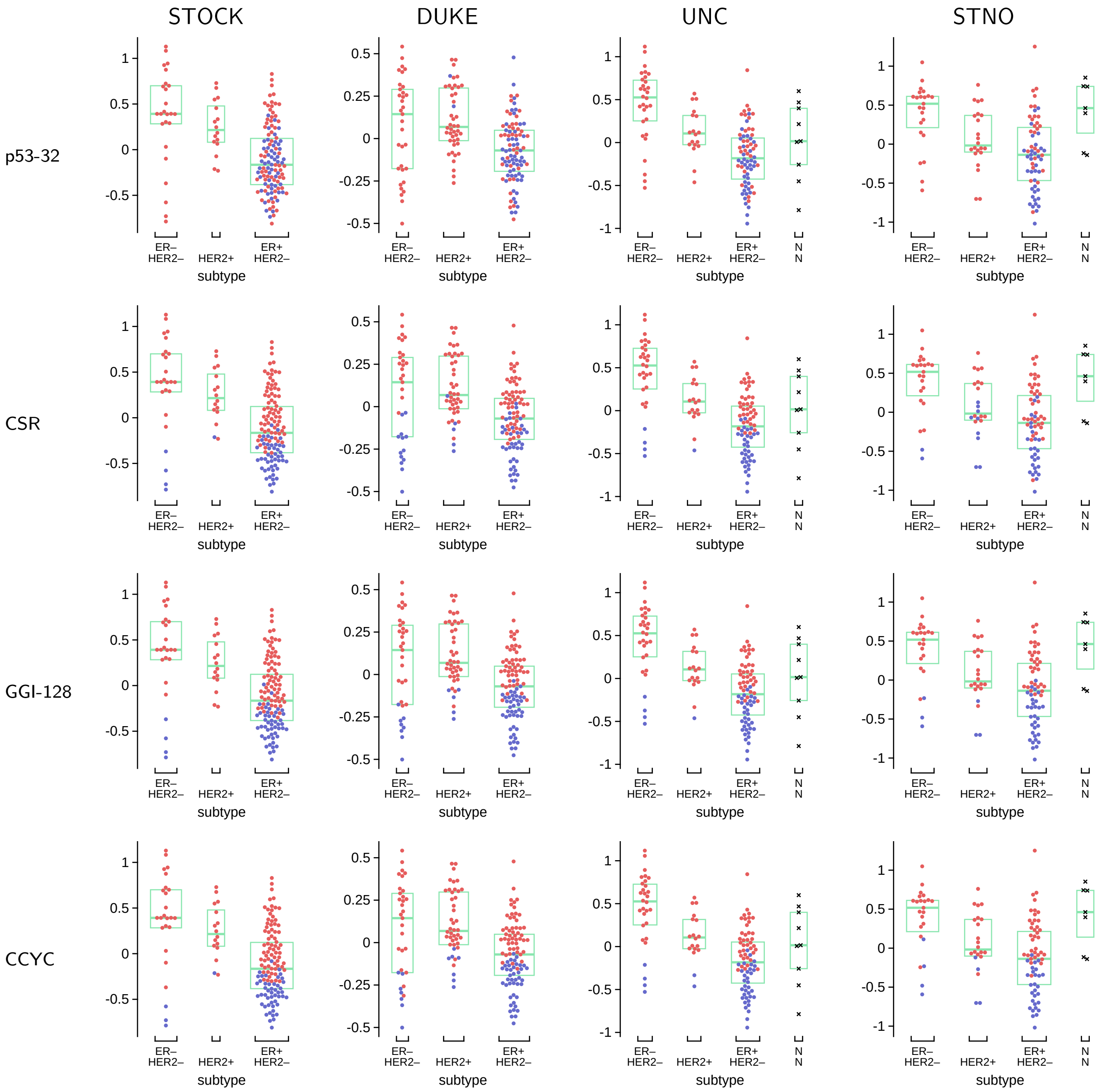

All vertical axes correspond to proliferation score.

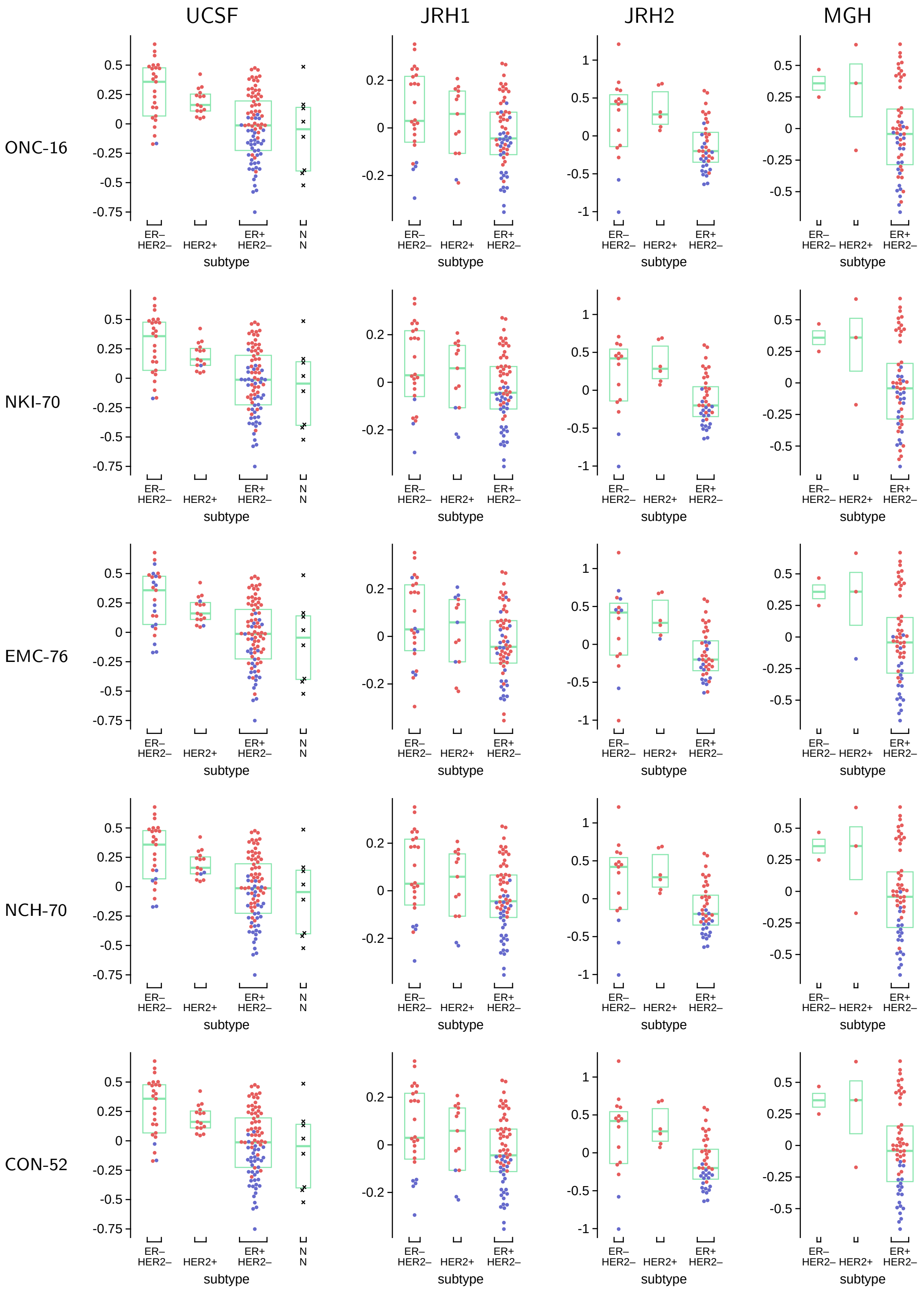

All vertical axes correspond to proliferation score.

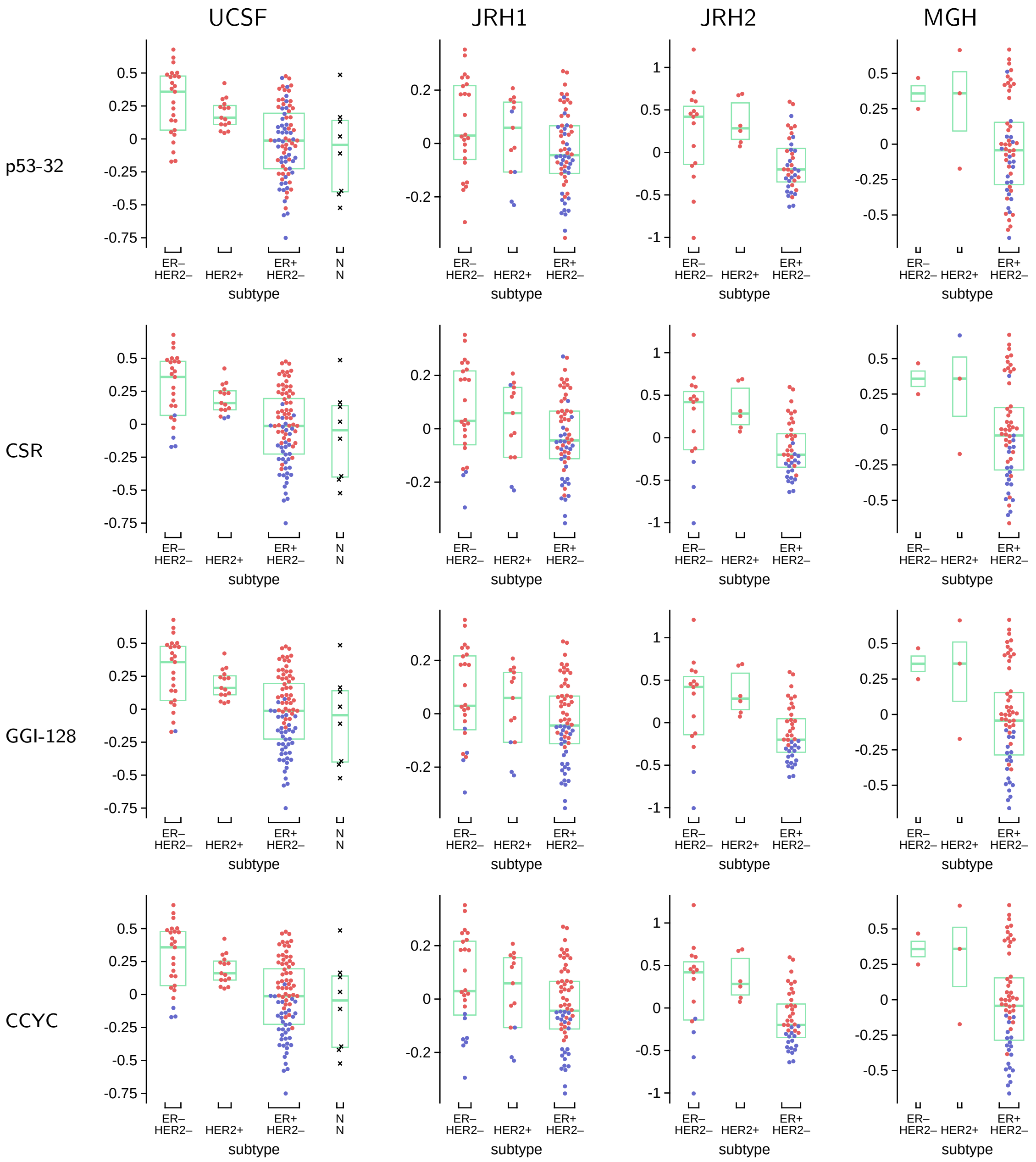

Combined prediction by pairs of signatures

Every distinct pair of signatures were combined in the following way:

1. Their continuous prediction scores were used as explanatory variables in Cox regression (stratified by dataset).
2. The fitted coefficients were used as weights in linear combination of the two prediction scores to produce the combined prediction score
3. The combined scores were used to rank patients. 33% in each dataset were classified as low-risk.

Figure 6. The results for the 36 pairs are shown below as the black bars to the right of the individual signatures. The identity of each combination is not shown (the ordering from left to right is [ONC-16 + NKI-70], [ONC-16 + EMC-76p], and so on), because we only want to demonstrate that their hazard ratios and survivals are similar and within the confidence interval of the some individual signatures. Note that the combined performance may be slightly biased upward because the weights are estimated from the same data. Even so, the improvement is not clinically substantial.

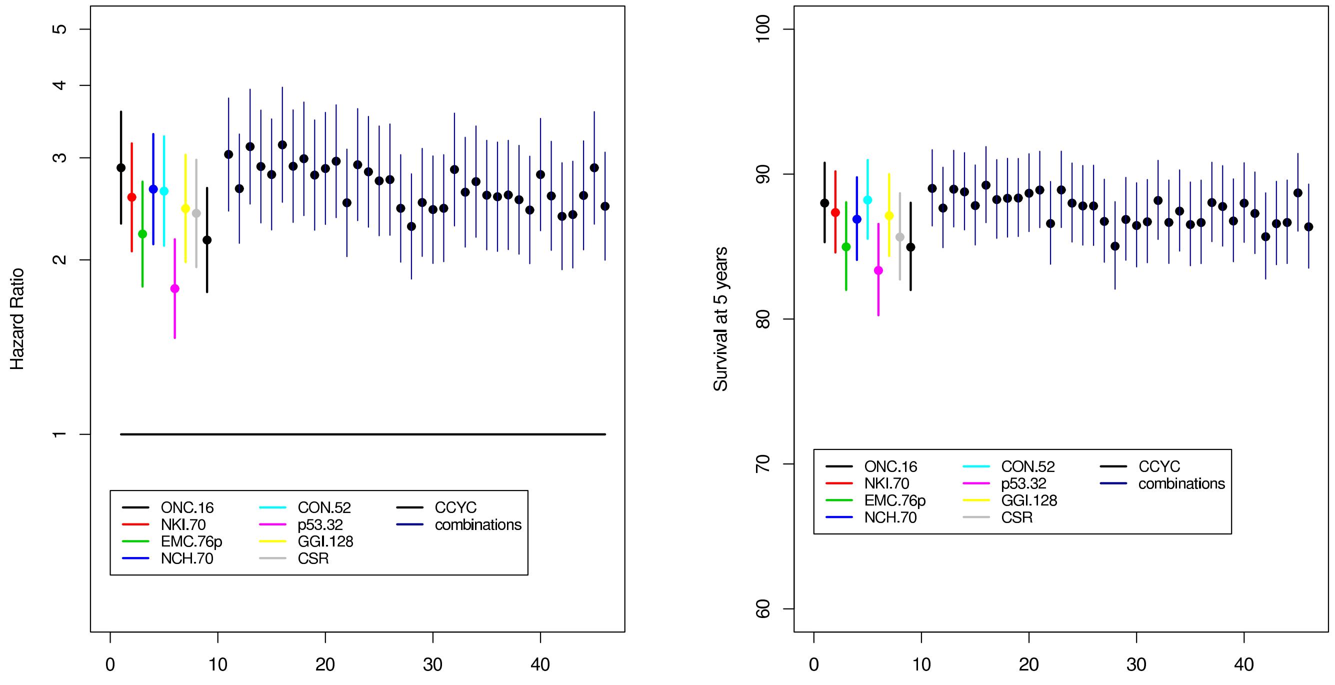

Supplement: Additional file 2 — Supplementary results. Supplementary Results including Supplementary Figures 1–5 and the results from the 'Combined prediction by pairs of signatures' (Supplementary Figure 6). [file bcr2124-S2.pdf]
